# Auditory inputs modulate intrinsic neuronal timescales during sleep

**DOI:** 10.1038/s42003-023-05566-8

**Published:** 2023-11-20

**Authors:** Philipp Klar, Yasir Çatal, Stuart Fogel, Gerhard Jocham, Robert Langner, Adrian M. Owen, Georg Northoff

**Affiliations:** 1 Faculty of Mathematics and Natural Sciences, Institute of Experimental Psychology, Heinrich Heine University of Düsseldorf, Düsseldorf, Germany; 2 Institute of Neuroscience and Medicine, Brain & Behaviour (INM-7), Research Centre Jülich, Jülich, Germany; 3 The Royal’s Institute of Mental Health Research & University of Ottawa, Brain and Mind Research Institute, Centre for Neural Dynamics, Faculty of Medicine, University of Ottawa, 145 Carling Avenue, Room 6435, Ottawa, ON K1Z 7K4 Canada; 4 Sleep Unit, University of Ottawa Institute of Mental Health Research at The Royal, K1Z 7K4 Ottawa, ON Canada; 5 Institute of Systems Neuroscience, Medical Faculty, Heinrich Heine University Düsseldorf, Düsseldorf, Germany; 6 Departments of Physiology and Pharmacology and Psychology, Western University, London, ON N6A 5B7 Canada; 7 Centre for Cognition and Brain Disorders, Hangzhou Normal University, Tianmu Road 305, Hangzhou, Zhejiang Province 310013 China

**Keywords:** Cognitive neuroscience, Sensory processing

## Abstract

Functional magnetic resonance imaging (fMRI) studies have demonstrated that intrinsic neuronal timescales (INT) undergo modulation by external stimulation during consciousness. It remains unclear if INT keep the ability for significant stimulus-induced modulation during primary unconscious states, such as sleep. This fMRI analysis addresses this question via a dataset that comprises an awake resting-state plus rest and stimulus states during sleep. We analyzed INT measured via temporal autocorrelation supported by median frequency (MF) in the frequency-domain. Our results were replicated using a biophysical model. There were two main findings: (1) INT prolonged while MF decreased from the awake resting-state to the N2 resting-state, and (2) INT shortened while MF increased during the auditory stimulus in sleep. The biophysical model supported these results by demonstrating prolonged INT in slowed neuronal populations that simulate the sleep resting-state compared to an awake state. Conversely, under sine wave input simulating the stimulus state during sleep, the model’s regions yielded shortened INT that returned to the awake resting-state level. Our results highlight that INT preserve reactivity to stimuli in states of unconsciousness like sleep, enhancing our understanding of unconscious brain dynamics and their reactivity to stimuli.

## Abstract (web summary)

A fMRI analysis of human participants during conscious and unconscious states provides evidence that intrinsic neuronal timescales preserve their reactivity to environmental stimuli during sleep across seven local networks and across the complete cerebral cortex.

## Introduction

The brain’s ongoing spontaneous activity measured in the resting-state comprises a range of timescales varying in length and power, labeled intrinsic neuronal timescales (INT) in the recent neuroimaging literature (see^1,2^ for reviews). INT are observed across all neuroimaging modalities, ranging from electrophysiological single-unit recordings in humans^3^ and non-human primates^4^ over invasive electrocorticography (ECoG)^5^ and non-invasive electroencephalography (EEG) recording^6,7^, to hemodynamic functional magnetic resonance imaging (fMRI) scans^8–12^. Beyond resting-state studies, stimulus- and task-related studies under conscious wakefulness showed that INT can be modulated by the processing of stimuli, such as audiovisual^13^ and visual^14^ movie clips, auditory language processing^15,16^, and memory-related processes^17^. These studies investigated stimulus-induced INT modulation during conscious wakefulness. While present discussions of brain dynamics often focus on the neuronal correlates of consciousness (see^18,19^ for reviews), in line with INT analyses under conscious wakefulness, INT during states of unconsciousness like sleep remain relatively understudied (see refs. ^8,20^ for two paradigmatic exceptions).

The question remains if the observed INT modulation by stimuli during conscious wakefulness is preserved during a primary unconscious state like sleep. Our fMRI analysis aimed to investigate this question by comparing INT in a sleep resting-state and during sleep with concurrent auditory stimulus presentation. Two recent studies investigated changing INT lengths during unconscious states. Specifically, an EEG study^20^ observed a prolongation of INT from non-rapid eye movement (NREM or N) 1 to N3 sleep. An fMRI study^8^ observed abnormally prolonged resting-state INT in light and deep propofol-induced anesthesia. Concerning our aim, these observations leave open two questions. First, do INT also show prolongation during sleep in fMRI recordings? Second, in the case of prolonged INT during sleep, can significant stimulus-induced INT modulations still occur during this primary unconscious state? If INT would display significant stimulus-induced modulation during sleep, INT adaption to environmental stimuli could represent a necessary albeit non-sufficient predisposition for consciousness, enhancing our understanding of the transition from unconscious to conscious brain dynamics.

In neuroimaging, INT lengths are often measured by the electrophysiological or hemodynamic signal’s temporal autocorrelation (AC)^2,4,10–12,21^. The autocorrelation function is a dimensionless statistic that measures the degree of potentially repeating patterns, namely temporal correlations, in a time-series^22^. The autocorrelation window (ACW) is a specification of the autocorrelation function by a specifically chosen time lag for extracting one parameter from the autocorrelation function, such as by selecting the autocorrelation function’s ACW 50 (the time lag at which the autocorrelation crosses *r* = 0.5)^10,13^ or the lag of the first zero crossing (*r* = 0), abbreviated as ACW 0^1,7,23,24^. Besides investigating the temporal AC, we measured the median frequency (MF) that divides the power spectral density into two halves with an equal area under the curve^8,25,26^. The Wiener–Khinchin theorem connects AC with spectral contents^27^ measured via the MF. The AC can exhibit long frequency cycle durations that are observable in the frequency-domain^13,20^, where AC prolongation corresponds to a decrease in MF and vice versa. The MF can consequently index that longer INT in unconsciousness during sleep (compared to conscious wakefulness) are supported by a higher power in the low-frequency spectral content.

**Fig. 1:**
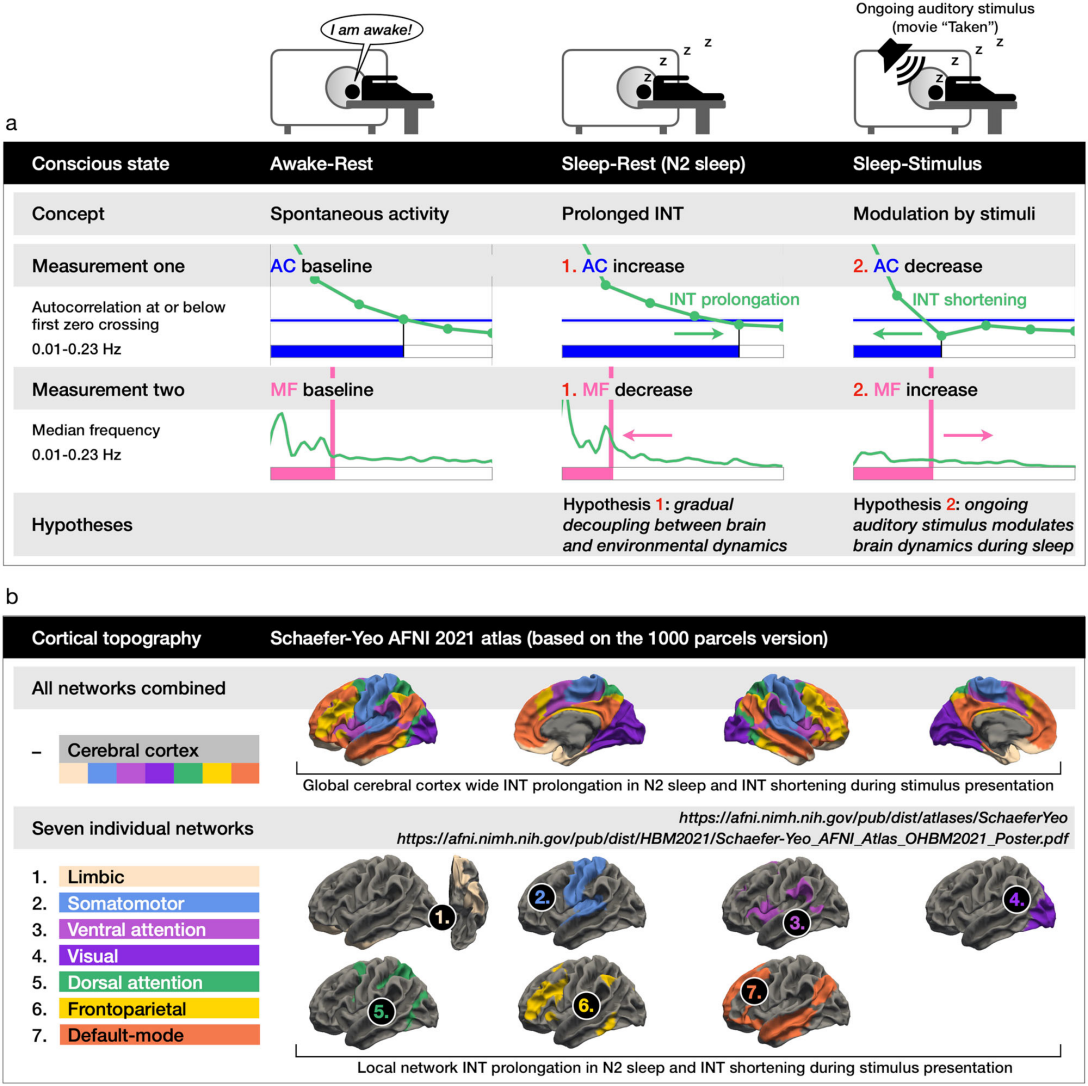
Overview of the two hypotheses and cortical topography. **a** Two analyzed measurements are the temporal autocorrelation (AC) and median frequency (MF). **b** Updated version of the Yeo seven network cortical parcellation, the Schaefer–Yeo AFNI 2021 atlas based on the 1000 parcels version (see section “Seven networks and cerebral cortex topography” for details). We investigated the local seven networks and their combination into a global level comprising the complete cerebral cortex.

We tested two hypotheses as displayed in Fig. 1a. Hypothesis one concerns the Awake-Rest vs. Sleep-Rest (N2 sleep) comparison, and hypothesis two the Sleep-Rest vs. Sleep-Stimulus (auditory stimulus presentation during sleep) comparison. Furthermore, we included a large-scale biophysical model^28^ that aimed to replicate the empirical results in simulations to validate the reactivity of INT and MF, where a sine wave simulated the extrinsic stimulus.

Hypothesis one (Awake-Rest vs. Sleep-Rest): Compared to Awake-Rest, we hypothesized a prolongation of INT during unconsciousness in Sleep-Rest, corresponding to increasing AC and decreasing MF, with the latter indexing a power shift towards slower frequencies. This hypothesis rests on previous EEG observations in the frequency band 1–80 Hz that showed INT prolongation under unconsciousness in sleep^20^, unresponsive wakefulness syndrome^20,24^, and propofol-induced anesthesia^20^. In the hemodynamic infra-slow frequency band (0.01–0.1 Hz), one fMRI study showed INT prolongation during propofol-induced anesthesia^8^, leaving open the question if sleep also prolongs INT in fMRI. Following these EEG and fMRI results and given that INT modulate the processing of environmental stimuli^1,13,14,28–30^, which is reduced during N2 sleep^31,32^, we hypothesized INT prolongation in Sleep-Rest, accompanied by a power shift towards slower frequencies indexed by MF.

Hypothesis two (Sleep-Rest vs. Sleep-Stimulus): Regarding the presentation of a continuous auditory stimulation for 5 min 12 s at the end of each participant’s sleeping session, we hypothesized INT shortening in Sleep-Stimulus, as compared to Sleep-Rest. A shortening of INT in response to the auditory stimulus would correspond to decreasing AC and increasing MF levels. The second hypothesis concerning sleep can extend previous study results that demonstrated INT modulation by environmental stimuli under conscious wakefulness^1,9,15–17,29^. We also base the second hypothesis of shortened INT across the cerebral cortex in response to the ongoing auditory stimulus on the following theoretical consideration: Two previous fMRI analyses by our group^33,34^ observed a cerebral cortex-wide and uniform response to an auditory stimulus, measured via two variables in the frequency-domain, namely the power-law exponent (the spectral slope in the logarithmic power spectrum) and mean frequency. More precisely, in the assessed slow event-related design with inter-trial intervals of 52–60 s^33^ and 15.5–25.5 s^34^, the BOLD signal showed a shift towards slower dynamics by increased power in slower and reduced power in faster frequencies, corresponding to a higher spectral slope and reduced MF that should, in turn, correspond to prolonged INT. Moreover, this uniform response of the two variables also occurred in the same seven individual networks investigated in this sleep fMRI analysis (see Fig. 5a in ref. ^34^).

Based on the above, we hypothesized that in response to ongoing naturalistic inputs (contrary to a slow event-related design), the brain potentially shows the opposite behavior in its stimulus-induced activity, that is, increased power in faster frequencies while decreasing power in slower frequencies, thus shortening INT in the same seven networks and the complete cerebral cortex. Shortened INT in response to the auditory stimulus (compared to Sleep-Rest) can potentially be related to the naturalistic stimulus’ variability that induces higher brain variability even during sleep, where increased BOLD variability results in shortened autocorrelation or INT. However, since we could not explicitly analyze the stimulus to test this additional hypothesis, we refrained from actually investigating this idea in the current analysis and left it for further research.

Furthermore, a recent study investigating conscious wakefulness demonstrated that a stimulus-induced state induced higher degrees of topographic similarity across cerebral cortex parcels than the resting-state^35^. Following this finding, we hypothesized a higher topographic similarity between cortical parcels during Sleep-Stimulus. More precisely, we hypothesized a more uniform distribution of INT across the parcels in each of the seven functional connectivity-based Yeo networks^36^ and in the global cerebral cortex parcels compared to the Sleep-Rest state (Fig. 1b displays the seven networks). We analyzed the topographic similarity via Fisher *Z* transformed pairwise Pearson correlation matrices of the seven networks’ and cerebral cortex’ cortical parcels^36^. We predicted higher correlations for Sleep-Stimulus compared to Sleep-Rest. Recent fMRI results highlighted significant global or brain-wide activity changes in unconscious states^37,38^ and thus provided a rationale for additionally analyzing the complete cerebral cortex beside the seven individual networks. Following the seven-network hypothesis, we likewise hypothesized brain-wide INT prolongation and decreased MF in Sleep-Rest compared to Awake-Rest, as well as INT shortening and increased MF in Sleep-Stimulus compared to Sleep-Rest. Figure 1a displays an overview of our functional MRI analysis’ two hypotheses, and Fig. 1b displays the investigated cortical topography.

In short, our findings revealed prolonged INT and decreased MF from the awake resting-state to the N2 sleep resting-state, and (2) INT shortened while MF increased during the auditory stimulus based on a movie clip in sleep. The biophysical model supported these results by demonstrating prolonged INT in slowed neuronal populations that simulate the sleep resting-state compared to an awake state.

## Results

We statistically compared two investigated states: (1) Awake-Rest vs. Sleep-Rest and (2) Sleep-Rest vs. Sleep-Stimulus. The Sleep-Rest state denotes N2 sleep, and Sleep-Stimulus refers to the auditory stimulus during sleep. The presentation of the AC and MF results is structured as follows: First, we present the local seven networks results, followed by the global cerebral cortex results. Second, we display the pairwise Pearson correlation matrices for the seven networks and the cerebral cortex, followed by the biophysical model results. Finally, we show the results of control analyses I and II.

Since not all participants remained fully asleep during the entire auditory stimulus presentation, even though no participants consciously recalled hearing the auditory stimulus at the end of the scanning session, we separately analyzed the AC and MF measurements in those four participants who remained fully asleep during the entire auditory stimulus presentation. We examined if these four participants showed the same result patterns as observed for all 17 participants.

### AC: local changes in seven networks

**Fig. 2:**
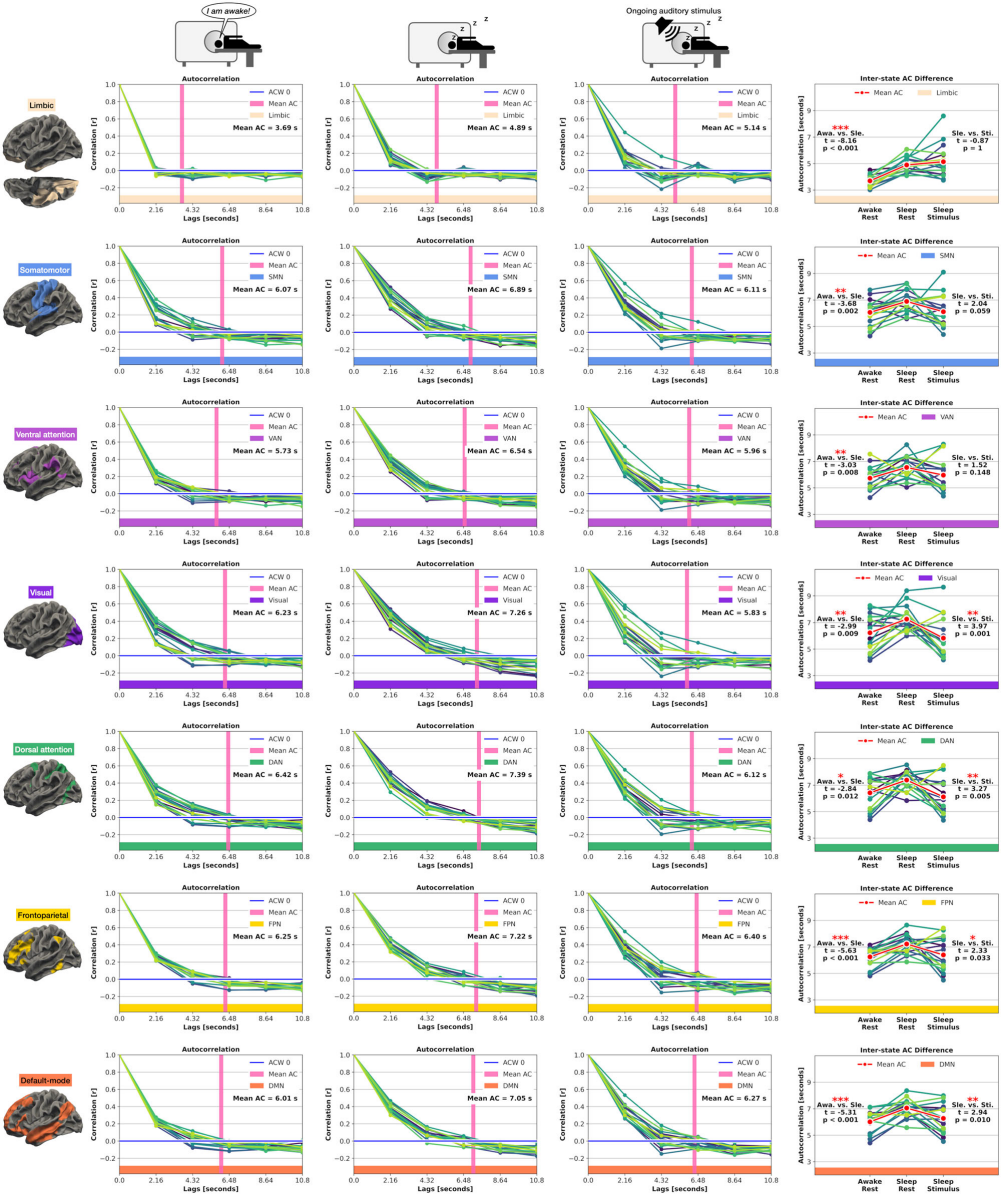
Temporal autocorrelation (AC) results for the seven networks in Awake-Rest, Sleep-Rest, and Sleep-Stimulus. Each line represents one participant, and the thicker red line represents the mean across participants. The horizontal blue line represents the first autocorrelation function’s first zero crossing, and the vertical pink line represents the voxel-based mean AC across all participants. (Awa, Awake-Rest; Sle, Sleep-Rest; Sti, Sleep-Stimulus; Statistics, Student’s paired *t* Test; significance asterisks, *p < 0.05, **p < 0.01, ***p < 0.001; Bonferroni correction = *p* values multiplied by two; *n* = 17 participants).

First, we analyzed if INT exhibit a significant prolongation in sleep, that is, in Sleep-Rest compared to Awake-Rest. Potentially prolonged INT in Sleep-Rest can serve as a baseline if these prolonged INT would retain the capability for a significant stimulus-induced modulation in the Sleep-Stimulus state, subsequently tested in the Sleep-Rest vs. Sleep-Stimulus comparison. Awake-Rest vs. Sleep-Rest: We observed significantly increased AC lengths from Awake-Rest to Sleep-Rest in all seven networks (*t* ≥ −2.84, *p* ≤ 0.012). Sleep-Rest vs. Sleep-Stimulus: Except for the limbic network (*t* = −0.87, *p* = 1), the remaining six networks showed reduced AC lengths from Sleep-Rest to Sleep-Stimulus. Four networks, precisely the visual, dorsal attention, frontoparietal, and the default-mode networks, showed significantly reduced Sleep-Stimulus vs. Sleep-Rest AC lengths (*t* ≥ 2.33, *p* ≤ 0.033). Together, the AC underwent significant stimulus-induced modulation in four of seven networks during sleep. Figure 2 displays the seven network AC results.

### MF: local changes in seven networks

**Fig. 3:**
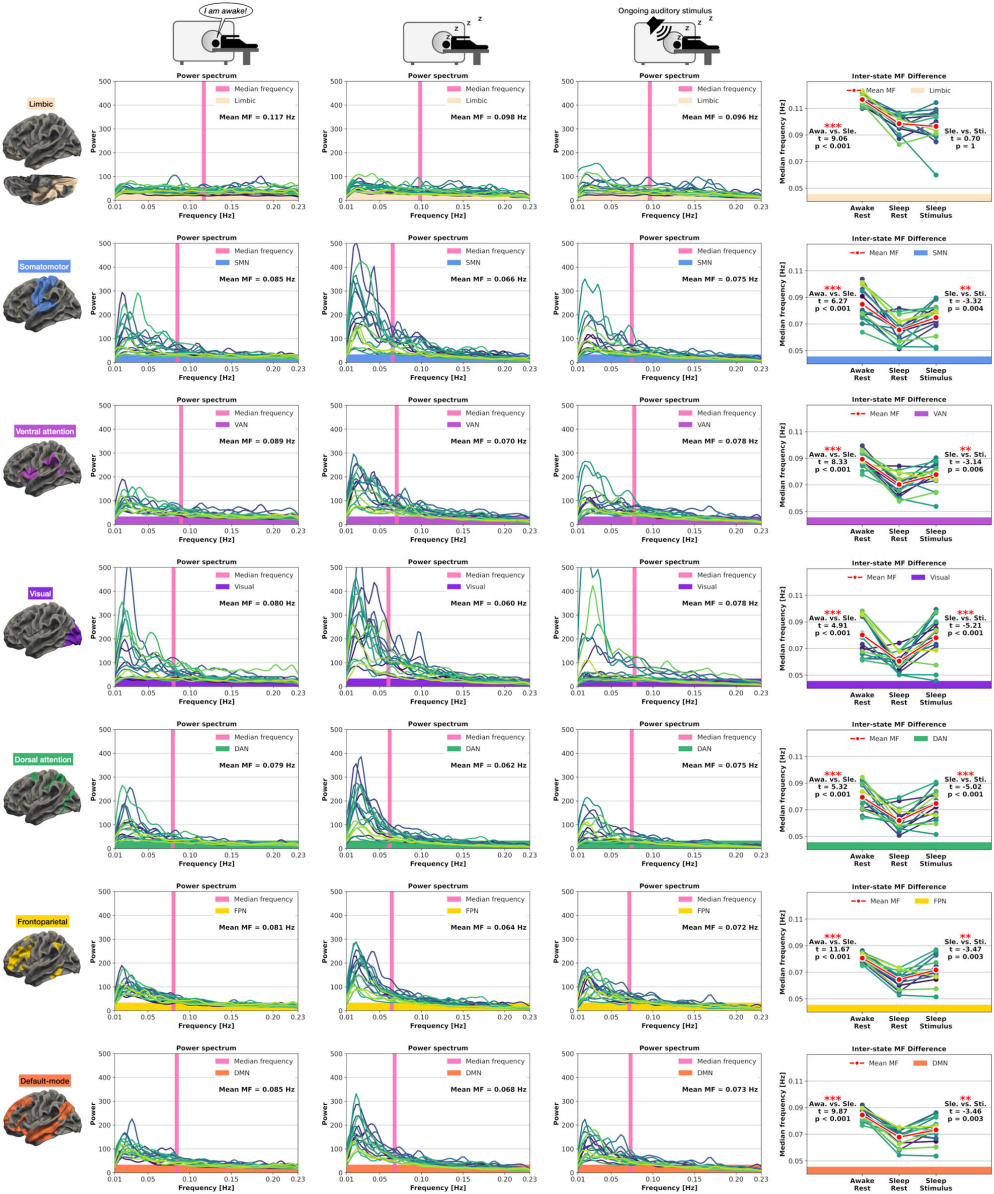
Power spectra and median frequency (MF) results for the seven networks in Awake-Rest, Sleep-Rest, and Sleep-Stimulus. Each line represents one participant, and the thicker red line represents the mean across participants. The vertical pink line represents the voxel-based average MF across all participants. (Awa, Awake-Rest; Sle, Sleep-Rest; Sti, Sleep-Stimulus; Statistics, Student’s paired *t* Test; significance asterisks, *p < 0.05, **p < 0.01, ***p < 0.001; Bonferroni correction = *p* values multiplied by two; *n* = 17 participants).

Given that slower frequencies can strongly modulate the AC length^13^, we also analyzed the MF in the frequency-domain in all three states to further validate the observed AC changes. Awake-Rest vs. Sleep-Rest: We observed increased power in slower frequencies and decreased power in faster frequencies in Sleep-Rest, corresponding to significantly decreased MF levels from Awake-Rest to Sleep-Rest in all seven networks (*t* ≥ 4.91, *p* ≤ 0.001). Sleep-Rest vs. Sleep-Stimulus: Except for the limbic network where the Sleep-Rest vs. Sleep-Stimulus MF did not significantly change (*t* = 0.7, *p* = 1), the remaining six networks showed increased MF from Sleep-Rest to Sleep-Stimulus (*t* ≥ −3.14, *p* ≤ 0.006). Together, the MF in the Sleep-Stimulus state increased in all seven networks compared to Sleep-Rest, demonstrating stimulus-induced modulation during sleep. Figure 3 displays the seven network MF results.

### AC: global changes in the cerebral cortex

Compared to conscious wakefulness, states of unconsciousness are not constrained to specific local network-based brain dynamics, but changes also occur in global or brain-wide activity, as compared to conscious wakefulness^37,38^. Accordingly, we analyzed AC in the cerebral cortex for the three investigated states to test if INT exhibit significant modulation across the entire cortical surface beyond specific and localized network changes from Awake-Rest to Sleep-Rest and Sleep-Rest to Sleep-Stimulus. Awake-Rest vs. Sleep-Rest: We observed significantly increased AC lengths from Awake-Rest to Sleep-Rest on the global level of the cerebral cortex (*t* = −5.89, *p* < 0.001). Sleep-Rest vs. Sleep-Stimulus: AC lengths significantly decreased when comparing Sleep-Rest vs. Sleep-Stimulus (*t* = 2.75, *p* = 0.014). The AC results followed our two hypotheses. First, INT showed a significant prolongation in Sleep-Rest vs. Awake-Rest in the local seven networks and global cerebral cortex analyses. Second, INT significantly decreased back or even below the Awake-Rest AC length during the presentation of the ongoing auditory stimulus. Together, these INT changes demonstrate the former’s brain-wide modulation during stimulus-induced inputs in sleep.

### MF: global changes in the cerebral cortex

**Fig. 4:**
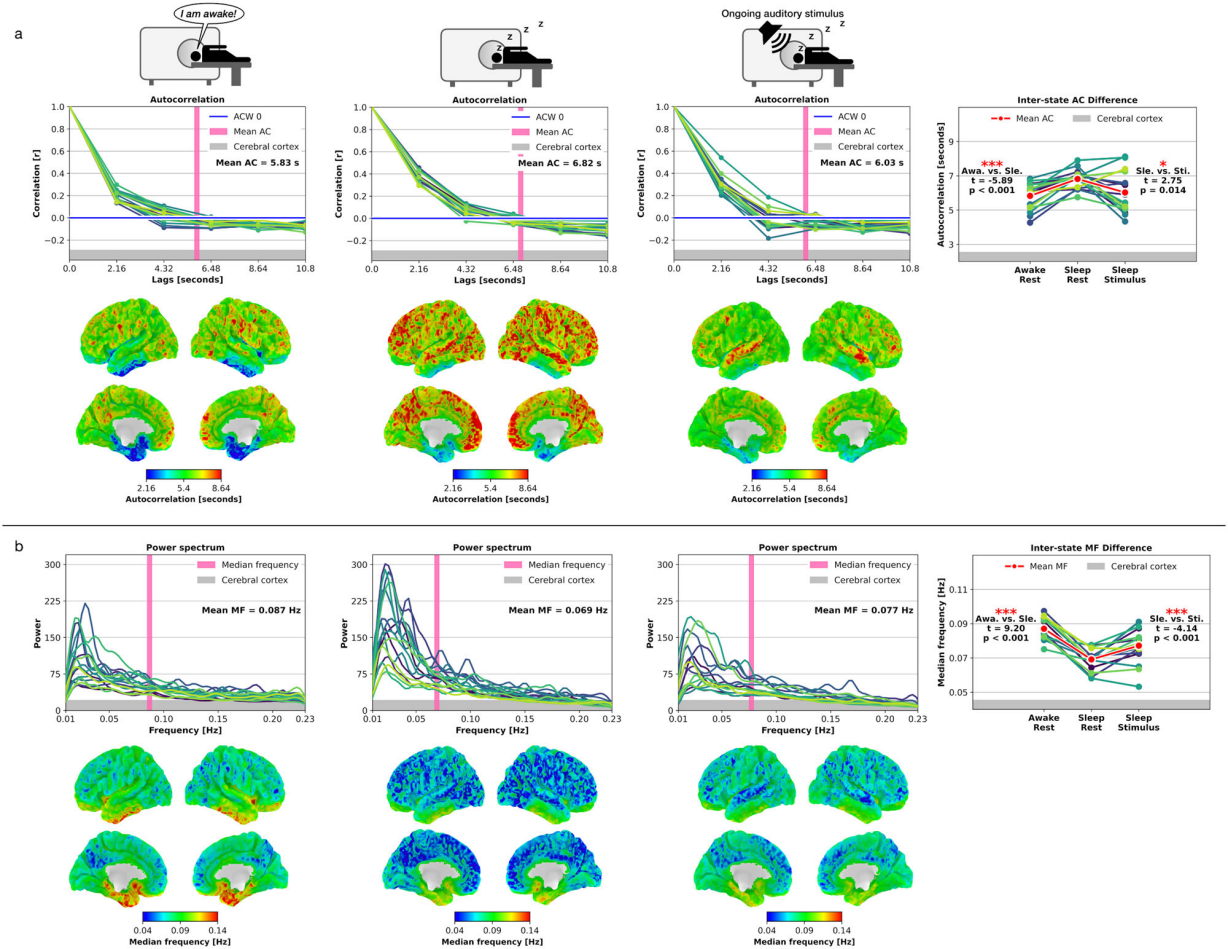
Temporal autocorrelation (AC) and median frequency (MF) results for the cerebral cortex in Awake-Rest, Sleep-Rest, and Sleep-Stimulus. **a** AC results. Each line represents one participant, and the thicker red line represents the mean across participants. The horizontal blue line represents the first autocorrelation function’s first zero crossing, and the vertical pink line represents the voxel-based mean AC across all participants. Cortical brain surfaces represent mean AC results across participants. **b** MF results. Each line represents one participant, and the thicker red line represents the mean across participants. The vertical pink line represents the voxel-based mean MF across all participants. Cortical brain surfaces represent mean MF results across participants. (Awa, Awake-Rest; Sle, Sleep-Rest; Sti, Sleep-Stimulus; Statistics, Student’s paired *t* Test; significance asterisks, *p < 0.05, **p < 0.01, ***p < 0.001; Bonferroni correction = *p* values multiplied by two; *n* = 17 participants).

Following the cerebral cortex AC analysis, we likewise computed the MF for the cerebral cortex to validate that global MF levels follow the observed global AC results. Awake-Rest vs. Sleep-Rest: We observed increased power in slower and decreased power in faster frequencies, corresponding to significantly decreased MF levels from Awake-Rest to Sleep-Rest on the global level of the cerebral cortex (*t* = 9.2, *p* < 0.001). Sleep-Rest vs. Sleep-Stimulus: In opposition to the Awake-Rest vs. Sleep-Rest comparison, the power spectra showed a shift in power from slower to faster frequencies when comparing Sleep-Rest vs. Sleep-Stimulus, corresponding to increased MF levels (*t* = −4.14, *p* < 0.001). Together, the MF results followed our two hypotheses and matched the AC results. First, MF levels decreased from Awake-Rest to Sleep-Rest in the seven networks and cerebral cortex. Second, the MF levels significantly increased in response to the ongoing auditory stimulus during Sleep-Stimulus on the local network and global cerebral cortex levels, with the only exception being the limbic network. Following the AC results, the MF results also demonstrated a global nature of stimulus-induced modulations in sleep. Figure 4a displays the global level cerebral cortex AC and Fig. 4b the MF results. Supplementary Note 4 provides additional information where Supplementary Tables 6 and 7 summarize the AC results and statistics, and Supplementary Tables 8 and 9 summarize the MF results and statistics.

### Topographic similarity of temporal AC: seven networks and cerebral cortex

To further track the auditory stimulus’ impact on the brain’s spatial topography beyond temporal changes measured in the time and frequency domain, we investigated the AC’s and MF’s topographic similarity by computing the Fisher *Z* transformed pairwise Pearson correlation matrices for the seven networks and the cerebral cortex in the three states analyzed here. The seven networks’ and cerebral cortex parcels allowed us to obtain the sets of Pearson correlation values so that every parcel yielded one Pearson correlation coefficient. As a result of the stimuli’s impact on primary unconscious brain dynamics during sleep, we predicted significantly different pairwise correlation distributions between Sleep-Rest and Sleep-Stimulus. We computed paired *t* Tests of the Pearson correlations for each network and the cerebral cortex. Additionally, we calculated the median pairwise correlation for all three states investigated.

**Fig. 5:**
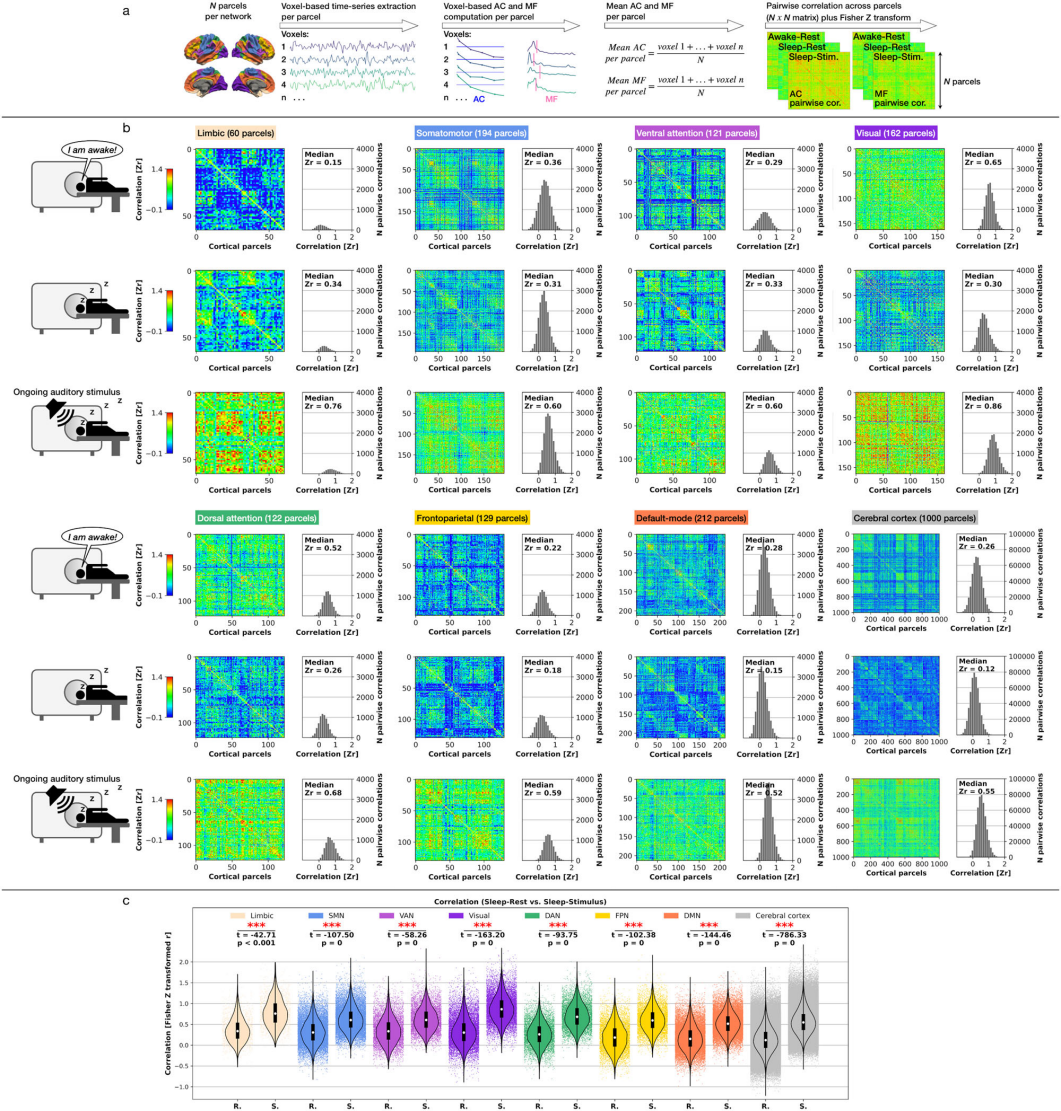
Pairwise Pearson correlation matrices for temporal autocorrelation (AC). **a** Schematic illustration of the applied steps for creation of the correlation matrices. **b** Seven networks and cerebral cortex correlation matrices. **c** Paired *t* Tests Pearson correlation values between Sleep-Rest (R) and Sleep-Stimulus (S); single data points represent voxel-based Pearson correlation values. Boxplot center point represents the median, boxes the interquartile range (IQR), and whiskers 1.5 × IQR (Statistics, Student’s paired *t* Test; significance asterisks, *p < 0.05, **p < 0.01, ***p < 0.001; *n* = 17 *p*articipants).

Seven networks: All seven networks showed the highest median correlation in Sleep-Stimulus compared to Awake-Rest and Sleep-Rest. All seven networks showed significantly higher correlations in Sleep-Stimulus compared to Sleep-Rest (*t* ≥ −42.71, *p* < 0.001). Cerebral cortex: The cerebral cortex overall showed an intermediate correlation in Awake-Rest (*r* = 0.26), the lowest correlation in Sleep-Rest (*r* = 0.12), and the highest correlation in Sleep-Stimulus (*r* = 0.55). Sleep-Stimulus yielded a significantly higher correlation than Sleep-Rest (*t* = −786.33, *p* = 0) for the cerebral cortex. The seven networks’ and cerebral cortex’s higher topographic similarity in Sleep-Stimulus compared to Sleep-Rest supports the notion that the auditory stimulation reveals a global reactivity of brain dynamics to (subconscious) environmental input that reaches beyond localized stimulus-induced modulations in specific networks or regions. Figure 5a displays a schematic illustration of the approach to compute pairwise correlation matrices, Fig. 5b displays the networks and cerebral cortex AC pairwise correlation matrices results, Fig. 5c the statistical results, and Supplementary Table 10 summarizes the results.

### Topographic similarity MF: seven networks and cerebral cortex

**Fig. 6:**
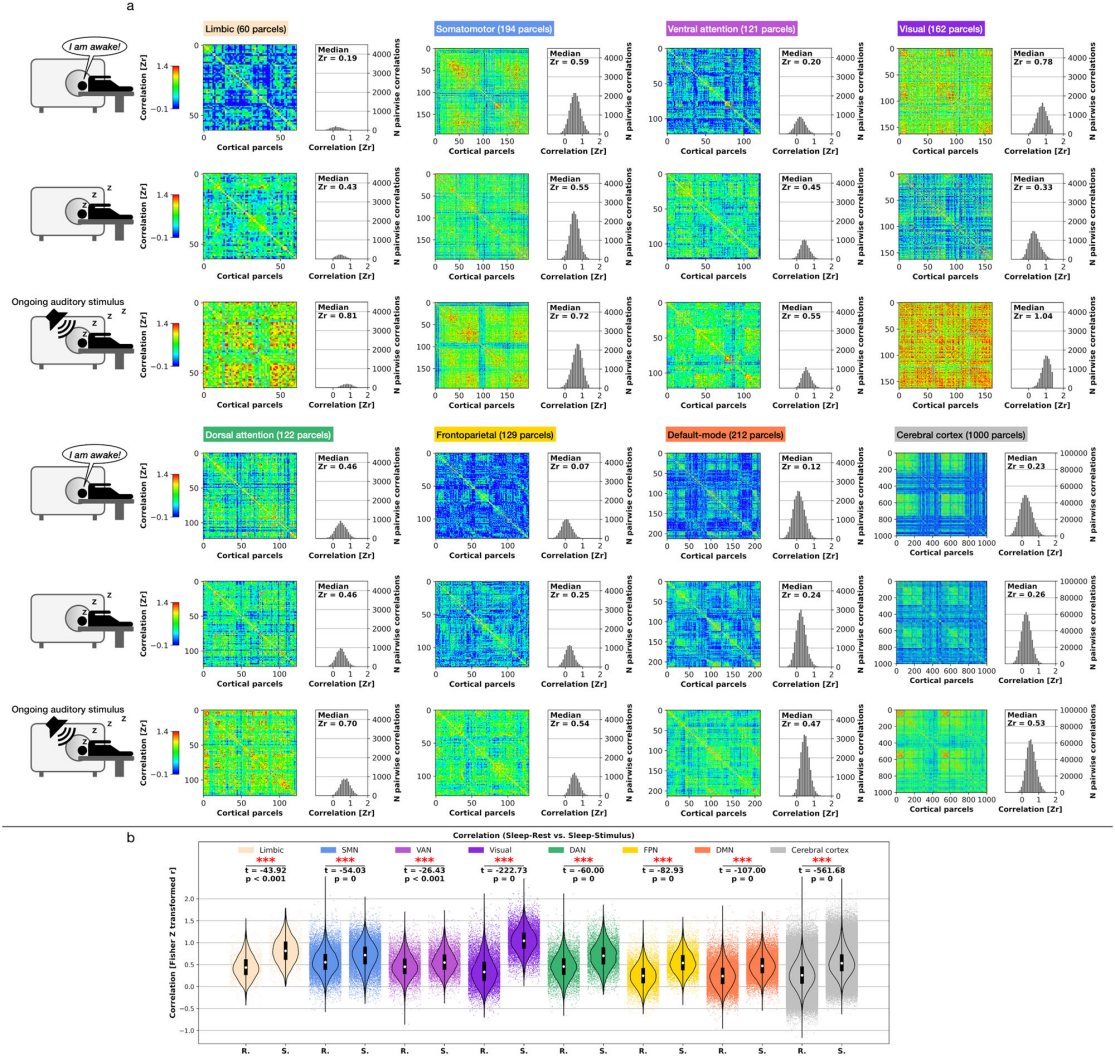
Pairwise Pearson correlation matrices for median frequency (MF). **a** Seven networks and cerebral cortex correlation matrices. **b** Paired *t* Tests Pearson correlation values between Sleep-Rest (R) and Sleep-Stimulus (S); single data points represent voxel-based Pearson correlation values. Boxplot center point represents the median, boxes the interquartile range (IQR), and whiskers 1.5× IQR (Statistics, Student’s paired *t* Test; significance asterisks, *p < 0.05, **p < 0.01, ***p < 0.001; *n* = 17 *p*articipants).

Seven networks: All seven networks showed the highest median correlation in Sleep-Stimulus compared to Awake-Rest and Sleep-Rest. All seven networks showed significantly higher correlations in Sleep-Stimulus compared to Sleep-Rest (*t* ≥ − 43.92, *p* < 0.001). Cerebral cortex: The cerebral cortex showed the lowest correlation in Awake-Rest (*r* = 0.23), a minimally higher correlation in Sleep-Rest (*r* = 0.26), and the highest correlation in Sleep-Stimulus (*r* = 0.53). Sleep-Stimulus yielded a significantly higher correlation than Sleep-Rest (*t* = −561.68, *p* = 0) for the cerebral cortex. Consequently, the topographic similarity results of MF perfectly followed the observed AC results by showing significantly increased topographic similarity in Sleep-Stimulus compared to Sleep-Rest. Figure 6a displays the networks and cerebral cortex MF pairwise correlation matrices, Fig. 6b the statistical results, and Supplementary Table 11 summarizes the results.

### Biophysical model

**Fig. 7:**
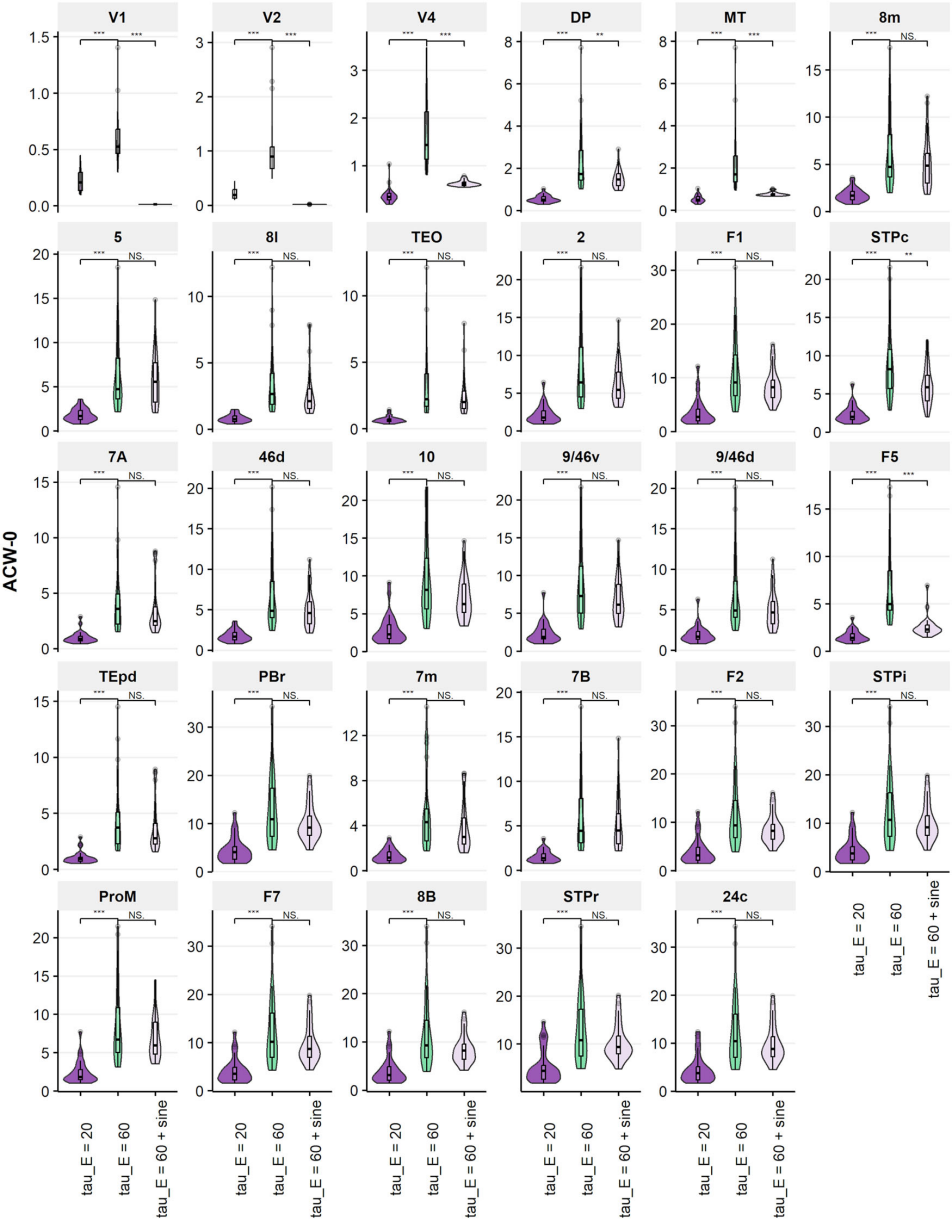
Large-scale biophysical model. We used parameters from ref. ^28^ to simulate the Awake state. The simulation of Sleep-Rest used increased time constant of excitatory regions. We applied a sine wave on top of white noise input for Sleep-Stimulus. The simulations were performed 30 times, the results show the distribution. (Statistics, Wilcoxon signed-rank test; significance asterisks, *p < 0.05, **p < 0.01, ***p < 0.001).

The empirical results demonstrated a significant stimulus-induced modulation of INT and MF during sleep. The results suggest that even prolonged INT during sleep remain the capability for stimulus-induced modulation, and we applied a realistic biophysical model^28^ to support our empirical results. The model simulated the three conditions, i.e., Awake-Rest, Sleep-Rest, and Sleep-Stimulus. For the Awake-Rest state, we used the model’s default parameters and provided white noise input to area V1. For Sleep-Rest, we changed the excitatory time constant from 20 to 60 ms, slowing down excitatory populations relative to inhibitory ones, and used the same white noise. For Sleep-Stimulus, we introduced a sine wave on top of white noise in the already slowed down model. Figure 7 displays the AC values for each region. Each violin shows the distribution of AC values across 30 simulations. Slowing down excitatory populations increased the AC values in all regions, akin to the empirical Sleep-Rest state. Conversely, most regions returned to their initial AC values under the presentation of a sine wave. The biophysical model results demonstrate that changes in the excitatory time constant can explain the observed INT prolongation in Sleep-Rest and the INT shortening in Sleep-Stimulus as observed in the empirical data.

In addition to our original input (a 25 Hz sine wave), we ran the same simulations with alternative input scenarios: combination of 3 sine waves with frequencies 25, 50, 75 Hz; 5 sine waves with frequencies 5, 25, 50, 75 and 100 Hz; and a waveform that goes between 20 and 100 Hz (shown in Supplementary Fig. 5) over the course of whole simulation. In all these different scenarios, we were able to get qualitative agreement with the results shown in Fig. 7. These results, presented in Supplementary note 3, are shown in Supplementary Figs. 3, 4, and 6, respectively. These results show that the results from our arbitrarily chosen 25 Hz scenario holds in different scenarios as well. Specifically, in the stimulation with the combination of 3 frequencies (Supplementary Fig. 3), 11 of the 29 stimulated regions significantly decreased their ACW 0 values with the stimulation, even with the higher excitatory time constant. Of interest, the regions 2, F1, 10 and F5 which are far from region V1 which we stimulated and outside the visual stream. In the combination of 5 frequencies (Supplementary Fig. 4), the ACW 0 values of 17 regions significantly decreased with stimulation. These included the non-visual stream regions F7, 7 m, 9/46d, 46d and among others. Finally, in the stimulation with 20 to 100 Hz changing input (Supplementary Fig. 6), ACW 0 values of 14 of the stimulated regions decreased including the regions outside the visual hierarchy ProM, 7B, F5, 46d, 2 and F1.

### Control analysis I: AC-MF correlation

We calculated the Pearson correlation coefficient and the coefficient of determination between AC and MF in the seven networks and the cerebral cortex presented in Supplementary Note 1. First, we aimed to evaluate if a negative and approximately linear relationship between both measurements empirically holds via the Pearson correlation coefficient. Second, we checked if the variation in MF can substantially predict the variation in AC via the coefficient of determination. Awake-Rest: All seven networks (*r* ≥ −0.63, *p* < 0.007) and the cerebral cortex (*r* = −0.82, *p* < 0.001) showed medium to high and approximately linear AC-MF correlations. Sleep-Rest: The seven networks (*r* ≥ −0.55, *p* ≤ 0.023) and cerebral cortex (*r* = −0.68, *p* = 0.002) showed medium to high and approximately linear AC-MF correlations. Sleep-Stimulus: The seven networks (*r* ≥ −0.82, *p* < 0.001) and cerebral cortex (*r* = −0.9, *p* < 0.001) showed high and approximately linear AC-MF correlations. Supplementary Fig. 1 displays and Supplementary Table 1 summarizes the AC-MF correlation results.

### Control analysis II: AC and MF in four participants fully asleep during the stimulus state

In the following, we present the control analysis comprising four participants that remained fully asleep during the entire stimulus presentation based on the simultaneous EEG recordings. We refrained from performing statistical tests due to the low number of participants. Instead, we inspected the results on a qualitative basis. We controlled if the four participants’ Awake-Rest to Sleep-Rest and Sleep-Rest to Sleep-Stimulus AC and MF results followed the seventeen participants’ results.

AC seven networks: Awake-Rest vs. Sleep-Rest: AC lengths increased from Awake-Rest to Sleep-Rest in all seven networks. Sleep-Rest vs. Sleep-Stimulus: Except for the limbic network, the remaining six networks showed shorter AC lengths in Sleep-Stimulus compared to Sleep-Rest. MF seven networks: Awake-Rest vs. Sleep-Rest: MF levels decreased from Awake-Rest to Sleep-Rest in all seven networks. Sleep-Rest vs. Sleep-Stimulus: Except for the limbic network, the remaining six networks showed higher MF levels in Sleep-Stimulus compared to Sleep-Rest. AC cerebral cortex: Awake-Rest vs. Sleep-Rest: AC lengths increased from Awake-Rest to Sleep-Stimulus. Sleep-Rest vs. Sleep-Stimulus: AC lengths decreased in the Sleep-Rest vs. Sleep-Stimulus. MF cerebral cortex: Awake-Rest vs. Sleep-Rest: The MF levels decreased from Awake-Rest to Sleep-Rest. Sleep-Rest vs. Sleep-Stimulus: The MF levels increased from Sleep-Rest to Sleep-Stimulus.

Overall, the four participants’ AC and MF results followed the observed results across the three investigated states for all seventeen participants. The four participants that remained entirely asleep during Sleep-Stimulus likewise showed shortened INT and increased MF in response to the auditory stimulus compared to Sleep-Rest. Supplementary note 2 includes a Figure of the results (Supplementary Fig. 2) where Supplementary Tables 2 and 3 summarize the AC results and Supplementary Tables 4 and 5 summarize the MF results.

## Discussion

Our empirical analysis showed INT prolongation and decreased MF in Sleep-Rest compared to Awake-Rest. We observed the reversed result pattern in Sleep-Stimulus, namely INT shortening and increased MF, where INT and MF approximately returned to their respective Awake-Rest levels. The biophysical model supported the empirically observed results by showing the same INT patterns across the three investigated states. Together, our results provide evidence for a significantly preserved capability for stimulus-induced modulation of INT in a primarily unconscious state like sleep. Additionally, the capability of INT to respond to (auditory) stimulation during unconsciousness reached beyond localized networks and included the complete cerebral cortex. The results complement recent observations of unconscious brain dynamics and their stimulus-induced modulation on the local network and global brain levels^8,18,20,37^.

Hypothesis one stated INT prolongation during N2 sleep (Sleep-Rest) compared to the awake resting-state. We observed INT prolongation on the local seven networks and global cerebral cortex topographic levels. Conversely, the MF decreased in N2 sleep, where power spectra displayed increased power in slower frequencies compared to the awake state. The observed spectral shift is in line with the well-known fact that, in addition to the occurrence of K-complexes and spindles, neuronal high-frequency and low-amplitude activity under conscious wakefulness shifts to a low-frequency and high-amplitude pattern under N2 and N3 sleep, as observed in electrophysiological^39–42^ and hemodynamic^8,43–45^ recordings of brain activity. The biophysical model emphasized the empirically observed INT prolongation during N2 sleep by showing that the increased excitatory time constant from 20 to 60 ms slowed down the model’s excitatory populations, resulting in increased AC values. Although we could not find any cellular neuroscience literature that compared the time constants between asleep and awake subjects, we hypothesize that this might be a generative model for our empirical findings, awaiting to be tested in future work. On the other hand, it should be mentioned that the possible set of models that can give the same results is huge and we can only test a few. For example, one might imagine that a change in excitatory-to-excitatory connection strengths can also lead to the phenomena observed. Nonetheless, our simulations provide at least one generative model for the data at hand. In addition, these results were observed again in different input scenarios as shown in Supplementary Figs. 3 and 4.

The modulation of the brain’s intrinsic spontaneous activity by extrinsic stimuli diminishes during N2 sleep, where long electrophysiological timescales in the delta band (0.5–4 Hz) dominate^41,46^ that suppress modulation by extrinsic sensory stimuli^31,32^. Compared to conscious wakefulness, unconsciousness in sleep leads to a gradual albeit not complete decoupling between neuronal and environmental dynamics^32^, including a ~20% reduced brain metabolism (blood flow) in NREM sleep^45–48^, potentially slowing down (prolonging) INT. The here observed INT prolongation and MF reduction during N2 sleep are also in line with EEG^20^, simultaneous EEG-fMRI^45^, and fMRI^8,43,44^ recordings that demonstrated increased low-frequency and high-amplitude/power spectral content in N2 sleep compared to conscious wakefulness, matching the results by our analysis. Paradigmatically, the functional MRI analysis by^41^ showed that high-frequency and low-power activity in sensory cortices under conscious wakefulness vanished in the NREM sleep stages, and Song et al.^45^ accordingly demonstrated highly increased EEG amplitude and BOLD power in slower frequencies during N2 sleep, corresponding to prolonged INT and reduced MF levels. Conversely, Song et al.^45^ observed low-amplitude/power in a mixed-frequency spectrum in the awake state, corresponding to the shortened INT and higher MF levels in Awake-Rest by our analysis.

Hypothesis two concerned the Sleep-Stimulus state. First, hypothesis two stated INT shortening due to modulation of the ongoing auditory stimulus (Sleep-Stimulus) compared to stimulus-free N2 sleep (Sleep-Rest). Second, hypothesis two assumed higher topographic similarity on the local seven networks and global cerebral cortex levels in Sleep-Stimulus than in Sleep-Rest. We observed INT shortening in the seven networks and in the cerebral cortex in Sleep-Stimulus. This INT modulation by the ongoing auditory stimulus in sleep during unconsciousness extends previous results that investigated stimulus- and task-related INT modulations constrained to conscious wakefulness^9,14,17,29,49–51^. The biophysical model supported the empirically observed INT shortening in Sleep-Stimulus where the introduction of a sine wave on top of white noise in the already slowed down model resulted in significantly shortened AC values.

Additionally, our finding of the INT’s global reactivity in the cerebral cortex to the auditory stimulus during sleep extents previous EEG^52–56^, simultaneous EEG-MEG^54^, and simultaneous EEG-fMRI^57–62^ studies that observed modulation of neuronal activity to sensory stimuli during sleep constrained to specific cortices investigated by other measurements, not including INT. The second aspect of hypothesis two, namely a global cerebral cortex modulation by the auditory stimulus during sleep, finds further support by the observation that not only the seven networks showed higher topographic similarity of INT during Sleep-Stimulus compared to Sleep-Rest, but that the cerebral cortex’s 1000 parcels version exhibited the highest topographic similarity during Sleep-Stimulus for both INT and MF.

We observed a close relationship between INT prolongation in Awake-Rest vs. Sleep-Rest and INT shortening in Sleep-Rest vs. Sleep-Stimulus with corresponding MF changes in both comparisons: MF decreases indexed INT prolongation in Sleep-Rest, while MF increases indexed INT shortening in Sleep-Stimulus. In this line, a recent fMRI^10^ investigated the relationship between INT and functional connectivity (FC) in the eyes-open resting-state in two datasets comprising 1139 and 10 participants, respectively. They analyzed the same seven network topography as in our analysis and applied global signal regression in the data preprocessing. They^10^ observed that INT have a close connection with functional networks on cortical (seven networks) and subcortical (striatum, thalamus, hippocampus, and cerebellum) levels where INT length systematically varied with FC intensity consistent with previous results^28,63^. More precisely, longer INT, measured by the ACW 50 in six of the seven networks leaving the limbic network out, corresponded to higher degrees of functional coupling, meaning that INT followed the known hierarchical–functional organization of higher-order anatomical structures^63–67^.

Furthermore, it has been demonstrated^10^ that inter-participant FC variability is related to inter-participant INT variability, with FC strength predicting INT length. Based on earlier findings^10^, combined with our observation of prolonged INT and decreased MF in Sleep-Rest, one theoretical inference is that relatively slower timescales substantially contribute to longer INT since slower timescales are related to global FC^10,28,63^. The high correlations between INT and MF for the seven networks and the entire cerebral cortex (Supplementary Fig. 1 and Supplementary Table 1) further underline that low-frequency and high-power spectral content substantially modulates INT length.

Finally, stimulus-induced INT modulations during sleep do not exclude the possibility that INT nonetheless serve as a neuronal predisposition of consciousness, as suggested by the Temporo-Spatial Theory of Consciousness (TTC)^68,69^. A neuronal predisposition of consciousness describes necessary but non-sufficient conditions for consciousness. The stimulus-induced INT modulation during sleep can represent a neuronal predisposition of consciousness. More precisely, INT must exhibit a dynamic capability for stimulus-induced modulation and, more generally, what we label temporo-spatial alignment to environmental stimuli^68,69^ so that consciousness can occur. If, in contrast, stimulus-induced INT modulation remains absent, consciousness would remain impossible, entailing that participants could no longer wake up to become conscious. Such a state is the propofol-induced loss of consciousness in anesthesia, where a recent fMRI analysis demonstrated the absence of task-induced modulation of brain dynamics^66^. Accordingly, our results suggest that INT modulation may play an important role in constituting unconscious brain dynamics that, subsequently, allow for a transition to conscious states mediated by other neuronal mechanisms. More generally, this indicates the importance of INT for a gray zone between unconscious and conscious states, as postulated by the TTC^68,69^. Our results extend previous observations of the spatial correlates of unconsciousness^8^ by including the temporal domain of brain dynamics.

We discuss limitations of our analysis in the following. The main limitation was that only four participants remained fully asleep throughout the entire auditory stimulus presentation at the end of the sleep scanning session. We applied control analyses for the four participants to ensure the same INT patterns across the three investigated states observed for all 17 participants. We replicated the INT results in the four participants obtained for the 17 participants. More precisely, the four participants showed prolonged INT in Sleep-Rest compared to Awake and shortened INT in Sleep-Stimulus compared to Sleep-Rest. Accordingly, the four participants’ MF results decreased in Sleep-Rest and increased in Sleep-Stimulus, as observed for all 17 participants.

The ongoing auditory stimulus only lasted 5 min 12 s, corresponding to a 145 sampling points time-series given a sampling rate (TR) of 2.16 s. The short time-series naturally comes with a lower signal-to-noise ratio than a time-series with more sampling points. Nonetheless, we refrained from analyzing the participants’ original full-length Awake and Sleep-Rest runs since we aimed to use identical time-series lengths for the three investigated states. Varying time-series lengths between Awake, Sleep-Rest, and Sleep-Stimulus would have likely affected INT and MF estimations differently in the three states, prohibiting sound methodological comparison between the three states.

Another limitation is that the limbic network showed slightly longer INT in Sleep-Stimulus than Sleep-Rest. This result does not follow the remaining six networks that, in turn, displayed shortened INT in Sleep-Stimulus. However, it is intriguing how consistently this pattern occurred across measures. It remains unsolved to what extent the limbic network’s slight INT prolongation in Sleep-Stimulus results from neuronal dynamics or if the relatively low signal-to-noise ratio of the limbic region impacted the results^34,69^. The limbic network is prone to signal dropout and high-frequency artifacts^70–72^, potentially leading to artifactual INT and MF results affected by non-neuronal or hemodynamic noise sources. By contrast, it is possible that during sleep, the limbic network is more internally-focused but processing information in a wake-like manner, consistent with studies demonstrating memory-related reactivation brain areas that output to the cortical aspects of the limbic network^73–76^.

Recent electrophysiological ECoG^77^ and EEG (see ref. ^78^ for a review), as well as simultaneous EEG-fMRI^44^ studies, provided empirical evidence of local sleep during conscious wakefulness, meaning that individual neuronal populations displayed slow wave activity indicative of N2 and N3 sleep while participants were awake. Conversely, other studies provided evidence of local wakefulness during sleep by observing brain regions that exhibited wake-like activity^79^ and that different brain areas showed sleep dynamics with various delays^80^, meaning that the awake-to-sleep transition did not uniformly occur in the brain. Together, these studies suggest that sleep can affect the brain in a heterogenous fashion: wakefulness and sleep can simultaneously emerge in different brain regions and request further analyses concerning local states of sleep and wakefulness. While our study demonstrated INT modulation by auditory inputs during sleep on both the local network and global cerebral cortex levels, we cannot rule out the possibility that the here investigated sleep states, namely Sleep-Rest and Sleep-Stimulus, exhibited local wakefulness in smaller areas of the cerebral cortex. It remains unknown to what extent small islands of local wakefulness impact or modulate INT prolongation during Sleep-Rest, and, conversely, INT shortening during Sleep-Stimulus. Such an investigation would have reached beyond our aim of exploring if INT significantly react to auditory inputs during sleep at all, and if this INT reactivity represents a brain-wide phenomenon.

Finally, although an additional analysis of the electrophysiological EEG data in the original EEG-fMRI study^62^ might lead to interesting results, we refrained from conducting this additional analysis for two primary reasons. First, we investigated specific regions originally obtained via a functional connectivity fMRI analysis^36^. It remains unclear and would require a new approach to properly link the obtained INT results in the seven investigated networks with time-series analyses for single EEG electrodes that have a very different spatial arrangement across the scalp’s surface. Second, we believe that the analysis of the obtained EEG data would be better suited for a potential future analysis that focuses on the electrophysiological properties of INT during wakefulness and sleep irrespective of the analyzed seven functional MRI networks and data, with a concentration on detailed EEG investigation that does not run into comprises when combining EEG and fMRI analyses into the same analysis or paper.

In this fMRI analysis, we investigated if the capability of INT for stimulus-induced modulation is also significantly preserved in an unconscious state like sleep. Indeed, INT showed prolongation during stimulus-free N2 sleep, while INT shortened during the presentation of continuous auditory stimulation in sleep, as compared to the previous stimulus-free N2 sleep. Besides the local level of seven networks, the auditory stimulus in sleep also modulated the global INT level of the cerebral cortex. Looking at the stimulation effects on the brain’s spatial surface, the cerebral cortex’s topographic organization underwent reorganization during the exposure to the auditory stimulus as manifested in higher topographic similarity compared to N2 sleep. The empirical and modeling results demonstrate the preserved capability of INT to be modulated by external stimuli in unconscious states like sleep. This INT modulation during sleep potentially represents a necessary albeit non-sufficient predisposition for consciousness.

## Methods

### Participants

The data collected for this analysis have been included in a separate previous simultaneous EEG-fMRI study addressing a different set of hypotheses^62^. The original EEG-fMRI study ensured that all participants provided informed consent and were financially compensated. This research was approved by the Western University Health Science REB^62^. Our analysis used data from 26 right-handed participants (male/female: 11/15; age mean/SD: 23.8/4.0 years) with no history of psychiatric, neurological, or sleep disorders. The study ensured that participants maintained a regular sleep schedule. Participants who met inclusion criteria were asked to wear an actigraph and to complete a sleep-wake log for one week before the scanning session. Participants were excluded if the actigraphy or sleep-wake logs suggested non-compliance with the study’s instructions. In the final analysis, participants slept for 44 minutes in the scanner, on average. We re-collected functional MRI data from 21 participants. Three participants had to be excluded from our fMRI analysis due to excessive head motion in the awake resting-state recording, leaving 18 participants for our final analysis. See the section Functional MRI preprocessing for exclusion criteria.

### Data acquisition

A Siemens 3 T Prisma scanner with a 64-channel head coil acquired whole brain scans via gradient-echo echo-planar imaging (EPI) (time repetition = 2160 ms, time echo = 30 ms, voxel size = 3.44 × 3.44 × 3 mm^3^, flip angle = 90°, slices = 40, inter-slice gap = 10%, matrix size = 64 × 64 × 40). T1 3D MPRAGE anatomical scans were acquired before the EEG-fMRI recordings (time repetition = 2300 ms, time echo = 2.98 ms, voxel size = 1 × 1 × 1 mm^3^, flip angle = 90°, matrix size = 256 × 256 × 176). The eyes closed awake resting-state run comprised 220 volumes (7 min 55 s), and the sleep run comprised up to 3333 volumes (120 min). Finally, the auditory ongoing stimulus, immediately presented prior to the end of the scanning session while participants were still asleep, comprised 145 volumes (5 min 12 s).

### Functional MRI awake resting-state and sleep recordings

The study used in-ear pneumatic headphones. An unrelated audio clip helped to set the headphone volume for each participant. The EEG-fMRI scanning session started at 9 pm with a T1 anatomical scan, followed by an 8 min eyes-closed awake resting-state recording. The sleep recording started at 10 pm with an average sleep onset latency of 8.16 ± 10.11 min, and participants were allowed to sleep for up to a maximum of 120 min during scanning. The auditory and ongoing stimulus, a part of the movie Taken was presented immediately before the end of each participant’s scanning session while the participant was still asleep. The movie excerpt consisted of a narrative of a conversation between a father and daughter that climaxes with the girl’s abduction, where the father subsequently delivers a threatening speech to the kidnappers. More precisely, participants were required to have had a period of at least 5 min of uninterrupted N2 or N3 sleep and to have remained asleep during the presentation of the ongoing auditory stimulus (5 min 12 s). At the end of the scanning session, ensuring headphone positioning remained intact during sleeping, participants were awoken and presented with an unrelated audio clip. Subsequently, the participants had to reply if they remembered hearing this auditory stimulus presentation. All participants asleep for the duration of the auditory stimulus had no recollection of hearing the stimulus. After the EEG-fMRI recordings, participants slept for the remainder of the night in the sleep laboratory.

### Functional MRI preprocessing

We performed preprocessing using AFNI (https://afni.nimh.nih.gov)^81^. The EEG-fMRI dataset comprised two runs, one awake resting-state, and one sleep run. The sleep run also included the auditory stimulus presentation at the end of each participant’s sleep scanning session. Before preprocessing the functional MRI data, we cut the raw awake and sleep runs to the same sampling points of 145 volumes matching the length of the ongoing auditory stimulus during sleep. We also cut the 5 min 12 s duration of the auditory stimulus presentation from the sleep run to preprocess and analyze the auditory stimulus presentation as a distinct Sleep-Stimulus run.

More precisely, we individually cut the sleep time-series for each participant to only include N2 sleep based on the EEG information that determined the sleep stages. The rationale for analyzing the N2 (light sleep) stage, instead of either the N1 (transitional sleep) or N3 (deep or slow wave sleep) stages, was that a maximum number of participants showed continuous or non-interrupted N2 sleep comprising at least 145 volumes. Only a few participants showed N1 or N3 stages lasting at least 145 volumes. Hence choosing the N1 or N3 stages for the sleep analysis would have drastically reduced the available number of participants for data analysis. The identical length after cutting the time-series also ensured that the investigated measurements, namely temporal autocorrelation and median frequency, were unaffected by otherwise varying time-series lengths or a varying number of volumes.

After cutting the raw data into three distinct time-series, namely Awake-Rest, Sleep-Rest, and Sleep-Stimulus, we individually preprocessed each of the three time-series applying the following steps in AFNI: (1) despiking and slice timing correction; (2) co-registration with high-resolution T1-weighted anatomical images; (3) non-linear spatial normalization of the anatomical scans into MNI152 2009c space and subsequent non-linear functional to anatomical alignment (normalization); (4) functional resampling to 3 × 3 × 3 mm^3^ voxels; (5) regression of linear and non-linear drift, and regression of local white matter signals to reduce non-neuronal noise^82^; (6) bandpass filtering using the frequency band of 0.01-0.23 Hz where the lower frequency was chosen to include at least 3 cycles for 0.01 Hz (within the 5 min 12 s time-series), and the upper frequency was constrained by the sampling rate (Nyquist frequency) since it has been shown that meaningful physiological data reaches beyond the often chosen 0.1 Hz limit^83–85^; (7) spatial smoothing using an 6 mm full-width at half-maximum isotropic Gaussian kernel; (8) motion censoring of volumes with Enorm (Euclidean norm of first differences of motion parameters) >0.35 mm or with an outlier fraction >10% within the whole-brain mask; (9) application of a third-order polynomial interpolation for censored volumes to preserve a continuous and intact time-series. We excluded participants exhibiting more than 7% censored volumes and participants with more than two successively censored volumes from further data analysis, leaving 17 from the re-collected 21 participants’ data for the final analysis.

### Statistics and reproducibility

We applied paired *t* tests in our analysis. We tested two parametric test assumptions^86^. First, we controlled the data’s approximate normality via the Shapiro–Wilk test within each state, i.e., in Awake-Rest, Sleep-Rest, and Sleep-Stimulus. Second, we tested the assumption of the data’s approximate homogeneity of variance via the Levene test. Results: All samples passed the Shapiro–Wilk and Levene tests, meaning that the samples’ significance always exhibited *p* > 0.05. Consequently, we did not reject the null hypotheses of normality and homogeneity.

We applied the Bonferroni correction to counterbalance the problem of multiple comparisons encountered in our analyses^86,87^. The Bonferroni correction counterbalances the multiple comparisons problem, namely the increased chance of obtaining false-positive comparisons. Instead of dividing the *p*-thresholds by the number of comparisons, paradigmatically *p* < 0.05/*n* where *n* is the number of comparisons, we multiplied the observed *p* values by *n*. The multiplication preserves the commonly used *p* thresholds for statistical significance of *p* < 0.05, *p* < 0.01, and *p* < 0.001 instead of lowering the *p* thresholds individually for statistical comparison. The multiplication of *p* values by *n* can result in *p* values exceeding 1; Bonferroni corrected *p* values that exceed one are displayed as *p* = 1 in our analysis.

Our analysis included two *t* Tests per network: (1) Awake-Rest vs. Sleep-Rest and (2) Sleep-Rest vs. Sleep-Stimulus. We accordingly applied a multiplication factor (Bonferroni correction) of two for *p* values to counterbalance the problem of possible false positives. The Bonferroni correction is conservative, and strictly controlling for false positives comes at the risk of false negatives.

### Seven networks and cerebral cortex topography

We assessed the brain’s cerebral cortex via the most recent version of the functional connectivity parcellation of 1000 young and healthy adults by^36^. The most recent version includes regions that match the MNI 2009c template instead of the MNI 2006 asymmetric template of the original publication by ref. ^36^. The updated version by AFNI improves structural contrast, allows better spatial contiguity, and offers better nonlinear alignment, consequently increasing correspondence across participants (see https://afni.nimh.nih.gov/pub/dist/HBM2021/Schaefer-Yeo_AFNI_Atlas_OHBM2021_Poster.pdf for an overview on the AFNI update for the Schaefer-Yeo atlas). The original Schaefer^88^ and Yeo^36^ atlases, including their update for AFNI, offer a variable number from 100 to 1000 parcellations. Based on the updated atlas distributed by AFNI (https://afni.nimh.nih.gov/pub/dist/atlases/SchaeferYeo/), we chose the 1000 parcellation version (500 per hemisphere) to create the seven Yeo networks: limbic, somatomotor (SMN), dorsal attention (DAN), ventral attention (VAN), visual, frontoparietal (FPN), and default-mode (DMN). Moreover, we chose the ribbon version of the updated Schaefer-Yeo atlas that primarily includes the cortical gray matter instead of the more liberal version that, in turn, has a higher degree of white matter. Besides investigating seven networks, we combined the seven networks into one region of interest that spans across the cerebral cortex, allowing the analysis at a global level.

### Temporal autocorrelation (AC) analysis

The autocorrelation function, a dimensionless statistic^22^, measures the degree of potentially repeating patterns in a time-series, where the sampling points’ earlier values can positively or negatively correlate with later values. Autocorrelation measurements find application in physiological time-series in general^89^ and in neuroimaging to estimate the length of intrinsic neuronal timescales^2,3,5,8,10^. Accordingly, higher compared to lower autocorrelation values can represent memory, the transmission or integration of information over time, and may reflect meaningful processing of internal and external stimuli. The autocorrelation window (ACW) specifies the individually chosen time lag for computing the autocorrelation statistic, where the ACW describes the window length of the autocorrelation function before the signal’s autocorrelation first reaches or drops below a specific threshold. Typical window lengths or thresholds applied in physiology^88^ or neuroimaging^2,7^ are the ACW 50 (where the autocorrelation crosses *r* = 0.5) and the ACW 0 (the first zero crossing of the autocorrelation function *r* = 0).

We analyzed the blood-oxygen-level-dependent (BOLD) temporal autocorrelation (AC) on a voxel-based level. Subsequently, we calculated the mean AC for each participant, allowing us to finally take the mean across all participants’ AC results. The AC formula (Eq. 1) at a given lag contains two ingredients, namely autocovariance (Eq. 2) and variance (Eq. 3). In these formulas, $N$ is the number of sampling points, $t$ is a time point in the time-series, $m$ is the lag, and $\bar{x}$ is the mean of the entire time-series (a constant). The autocorrelation for a specific lag is the autocovariance for that lag as standardized by the variance of the observations, namely the autocovariance divided by the variance^22^.

1 $${autocorrelation}=\frac{{autocovariance}}{{variance}}$$

2 $$autocovariance=\frac{1}{N}\mathop{\sum }\limits_{t=1}^{N-m}({x}_{t}-\overline{x})({x}_{t+m}-\overline{x})$$

3 $$variance=\frac{1}{N}\mathop{\sum }\limits_{t=1}^{N}{({x}_{t}-\overline{x})}^{2}$$

### Median frequency (MF) analysis

Further supporting INT observations, we measured the median frequency in the frequency-domain. The median frequency divides the area under the power spectral density curve into two halves of equal area^8,25,26^. The median frequency can reflect an additional index beside the AC supporting INT changes since INTs are related to long cycle durations of slower frequencies observable in the frequency domain^13,20^, where longer cycles allow better temporal integration of various stimuli across time. Accordingly, the MF can be used as an unbiased index of the extent of temporal integration of information in different conscious and unconscious states. To compute the MF, we transformed the time-series into the frequency domain on a voxel-based level via a Periodogram. We then computed the median frequency on the voxel-based level for each participant on the local level of the seven networks and on the global level of the cerebral cortex. Finally, we took the mean across all voxels’ median frequency results for each participant.

### Topographic similarity of AC and MF

We also asked to what extent the local seven networks and the global cerebral cortex exhibit a higher topographic similarity of AC and MF in Sleep-Stimulus compared to Sleep-Rest to understand the spatial uniformity of the information processing that potentially increases from Sleep-Rest to Sleep-Stimulus. To investigate this question, we computed the pairwise Pearson correlation using the networks’ single parcels of the updated Schaefer-Yeo 1000 parcels atlas by AFNI (https://afni.nimh.nih.gov/pub/dist/atlases/SchaeferYeo/). We calculated the pairwise Pearson correlation for the seven networks and cerebral cortex. First, we computed voxel-based AC and MF for each of the network’s or cerebral cortex’s respective parcels for every participant. Second, we calculated the mean AC and MF across each parcel’s voxel-based AC and MF results for every participant. Next, we computed the pairwise Pearson correlation of the *N* × *N* parcel matrix, where *N* is the number of the respective network’s or cerebral cortex’s parcels: limbic = 60, somatomotor = 194, ventral attention = 121, visual = 162, dorsal attention = 122, frontoparietal = 129, default-mode = 212, and cerebral cortex = 1000 parcels).

Using paired *t* Tests, we statistically controlled if the Pearson correlation distributions between Sleep-Rest and Sleep-Stimulus significantly diverged, based on the hypothesis of a higher topographic similarity in Sleep-Stimulus (compared to Sleep-Rest). Before performing t-tests for each network and the cerebral cortex, we applied the Fisher *Z* transform on the Pearson correlation values, i.e., the Fisher *r*-to-*Z* transform. While our statistical comparison focused on the *z*-transformed Pearson correlation distributions between Sleep-Rest and Sleep-Stimulus, we additionally calculated the median pairwise correlation for Awake-Rest, Sleep-Rest, and Sleep-Stimulus after leaving one triangular side out (to discard duplicates) and the diagonal line that divides the two triangular sides (to remove meaningless comparisons).

### Biophysical model

We used a large-scale biophysical model introduced by ref. ^28^ to further validate our empirical results. The model consists of the following differential Eqs. 4 and 5.

4 $${\tau }_{E}\frac{d}{{dt}}{v}_{E}^{i}=\,-{v}_{E}^{i}+{\beta }_{E}\left[\left(1+\eta {h}_{i}\right)\left({w}_{{EE}}{v}_{E}^{i}+{\mu }_{{EE}}{\varSigma }_{j=1}^{N}{{FLN}}_{{ij}}{v}_{E}^{\,j}\right)-{w}_{{EI}}{v}_{I}^{i}+{I}_{{ext},E}^{i}\right]_{+}$$

5 $${\tau }_{I}\frac{d}{{dt}}{v}_{I}^{i}=-{v}_{I}^{i}+{\beta }_{I}\left[\left(1+\eta {h}_{i}\right)\left({w}_{{IE}}{v}_{E}^{i}+{\mu }_{{IE}}{\varSigma }_{j=1}^{N}{{FLN}}_{{ij}}{v}_{E}^{\,j}\right)-{w}_{{II}}{v}_{I}^{i}+{I}_{{ext},I}^{i}\right]_{+}$$

In Eqs. 4 and 5$v$ is the firing rate, $\tau$ is intrinsic time constant, and *I*_*Ext*_ is the external input to the system governed by the slope β of the linear threshold f-I curve. *w* values are coupling parameters. μ is a fixed parameter that controls the strength of long-range excitatory input. ${FLN}$ (Fraction of Labeled Neurons) is the structural connectivity matrix based on a macaque study^89,90^. $E$ and $I$ correspond to excitatory and inhibitory, respectively; $i$ and $j$ denotes different regions. $h$ values were assigned to each area such that the difference in values predicts SLN according to a logistic regression function ${g}^{-1}$ shown in Eq. 6.

6 $${FLNij}={g}^{-1}({hi}-{hj})$$

Resulting $h$ values were normalized by dividing them to the biggest $h$. We used the exact same parameters as ref. ^28^. The region V1 was given Gaussian noise with 0-mean and unit variance. Every other region received Gaussian noise with 0-mean and an SD of 10^−5^. The model was simulated for 300 s using Euler–Maruyama method with a time-step of 1 ms. The first 50 seconds were discarded. To simulate the sleeping state, we changed excitatory time constant *τ*_*E*_ from 20 to 60 ms. On a 1-dimensional differential equation, this change would have been trivial, in the sense that it would only scale the time axis differently. However in the model where excitatory and inhibitory populations have different time constants (20 ms and 10 ms respectively), increasing time constant only for excitatory population would mean slowing down of excitatory populations relative to inhibitory ones. We simulated this modified model with the same Gaussian noises by resetting the random number generator. Finally, we applied a sine wave of 25 Hz frequency on top of same Gaussian noises to simulate sleeping condition with stimulus. The sine wave, as opposed to noise, has a finite timescale and non-zero information, akin to a stimulus. We calculated ACW-0 values the same way as empirical data on excitatory firing rates of each region. We performed 30 simulations for each scenario to get 30 ACW-0 values per ROI per state (Awake-Rest, Sleep-Rest, Sleep-Stimulus).

One might rightfully argue that the choice of 25 Hz in the simulation is arbitrary. Therefore, we ran the same simulations with the following scenarios: (i) combination of 3 sine waves with frequencies 25, 50, 75 Hz added on top of usual white noise input; (ii) combination of 5 sine waves with frequencies 5, 25, 50, 75 and 100 Hz added on top of usual white noise input (iii) a waveform that goes between 20 and 100 Hz over the course of whole simulation added on top of white noise input. The results of these additional analyses are in agreement with the original 25 Hz scenario and are presented in the supplementary material.

### Control analysis I: AC-MF correlation

We aimed to support temporal autocorrelation (AC) changes across the three investigated states with a second measurement, namely median frequency (MF). To control that a close and approximately linear relationship between AC and MF empirically holds, we computed the Pearson correlation coefficient and the coefficient of determination (*r*-squared) in the seven networks and cerebral cortex. The Pearson correlation coefficient calculates the strength and direction of a linear relationship between two variables. The coefficient of determination calculates how much of the variation in percent in one variable, i.e., AC, can be accounted for by the other variable, i.e., MF.

### Control analysis II: AC and MF in four participants fully asleep during the stimulus state

Besides analyzing AC and MF for the included 17 participants after preprocessing and excluding participants not meeting our exclusion criteria (see section Functional MRI preprocessing), we analyzed both measurements in four of the 17 participants that remained fully asleep during the entire ongoing 5 min 12 s auditory stimulus. The control analyses ensured that the same AC and MF results in the Sleep-Stimulus state, as observed for all participants, also apply to the four participants that remained fully asleep during the auditory presentation at the end of their respective scanning session.

### Reporting summary

Further information on research design is available in the Nature Portfolio Reporting Summary linked to this article.

### Supplementary information

Supplementary information

Description of Additional Supplementary Files

Supplementary data 1

Reporting summary

## Data Availability

The functional MRI dataset assessed in this analysis is available from the corresponding author upon reasonable request. The Source data for Figs. 2–7 can be found in Supplementary Data 1.

## Code availability

The code to replicate the biophysical (computational) model analysis is publicly available at https://github.com/duodenum96/sleep_paper^91^.
