# Supplementary material for: Auditory inputs modulate intrinsic neuronal timescales during sleep
Source: Commun Biol. 2023 Nov 20;6:1180. doi: 10.1038/s42003-023-05566-8 (PMC10661171; doi:10.1038/s42003-023-05566-8)
Supplement: Supplementary file 1 — Supplementary information [file 42003_2023_5566_MOESM1_ESM.pdf]

Supplementary information for "Auditory inputs modulate intrinsic neuronal timescales during sleep"

Supplementary note 1: AC-MF correlation

To control that a close and approximately linear relationship between AC and MF empirically holds, we computed the Pearson correlation coefficient (Pearson's r) and the coefficient of determination (r^2) in the seven networks and cerebral cortex. We observed linear and high (negative) relationships between AC and MF for the seven networks and cerebral cortex in the three investigated states (Awake-Rest, Sleep-Rest, and Sleep-Stimulus). Supplementary Figure 1 displays and Supplementary Table 1 summarizes the Pearson correlation coefficients and coefficients of determination (r-squared) between AC and MF.

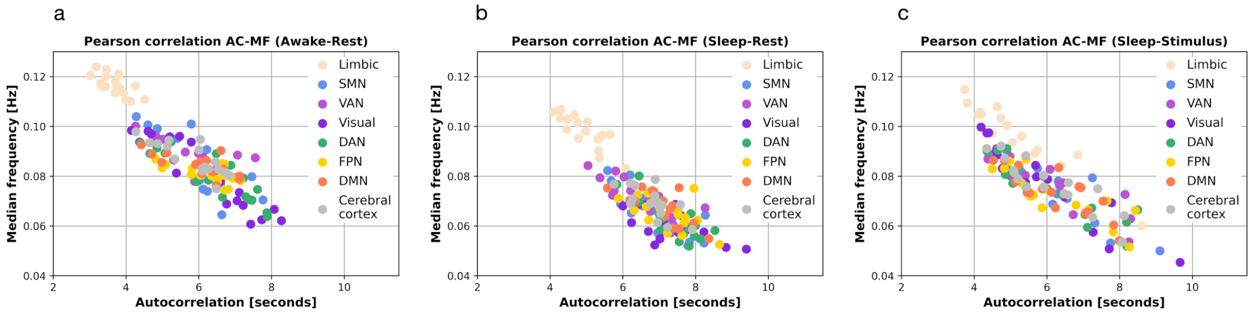

Supplementary Figure 1. Pearson correlation between temporal autocorrelation (AC) and median frequency (MF). Dots represent single subject AC-MF correlations. **a)** Awake-Rest. **b)** Sleep-Rest. **c)** Sleep-Stimulus. n = 17 subjects.

Supplementary Table 1
Pearson correlation between temporal autocorrelation (AC) and median frequency (MF)

		Limbic	SMN	VAN	Visual	DAN	FPN	DMN	Cerebral cortex
Awake-Rest	*r*-value	-0.70	-0.81	-0.69	-0.94	-0.88	-0.63	-0.71	-0.82
	r-squared	0.49	0.66	0.48	0.88	0.77	0.40	0.50	0.67
	p-value	0.001 **	< 0.001 ***	0.002 **	< 0.001 ***	< 0.001 ***	0.007 **	0.001 **	< 0.001 ***
Sleep-Rest	*r*-value	-0.85	-0.81	-0.82	-0.61	-0.80	-0.55	-0.72	-0.68
	r-squared	0.73	0.66	0.67	0.37	0.64	0.30	0.51	0.47
	p-value	< 0.001 ***	< 0.001 ***	< 0.001 ***	0.009 **	< 0.001 ***	0.022 *	0.001 **	0.002 **
Sleep-Stimulus	*r*-value	-0.94	-0.88	-0.88	-0.93	-0.91	-0.84	-0.82	-0.90
	r-squared	0.88	0.78	0.77	0.87	0.82	0.70	0.67	0.80
	p-value	< 0.001 ***	< 0.001 ***	< 0.001 ***	< 0.001 ***	< 0.001 ***	< 0.001 ***	0.002 **	< 0.001 ***

Significance asterisks, $p < 0.05$ *, $p < 0.01$ ** , $p < 0.001$ ***; n = 17 subjects. (SMN, somatomotor network; VAN, ventral attention network; DAN, dorsal attention network; FPN, frontoparietal network; DMN, default-mode network.)

Supplementary note 2: AC and MF in four subjects fully asleep during the stimulus state

Besides analyzing AC and MF for the included eighteen subjects, we additionally analyzed both measurements in four of the eighteen subjects that remained fully asleep during the ongoing 5 min 12 s auditory stimulus. Supplementary Figure 2 displays the results. Supplementary Tables 2 and 3 summarize the AC while Supplementary Tables 4 and 5 summarize the MF results.

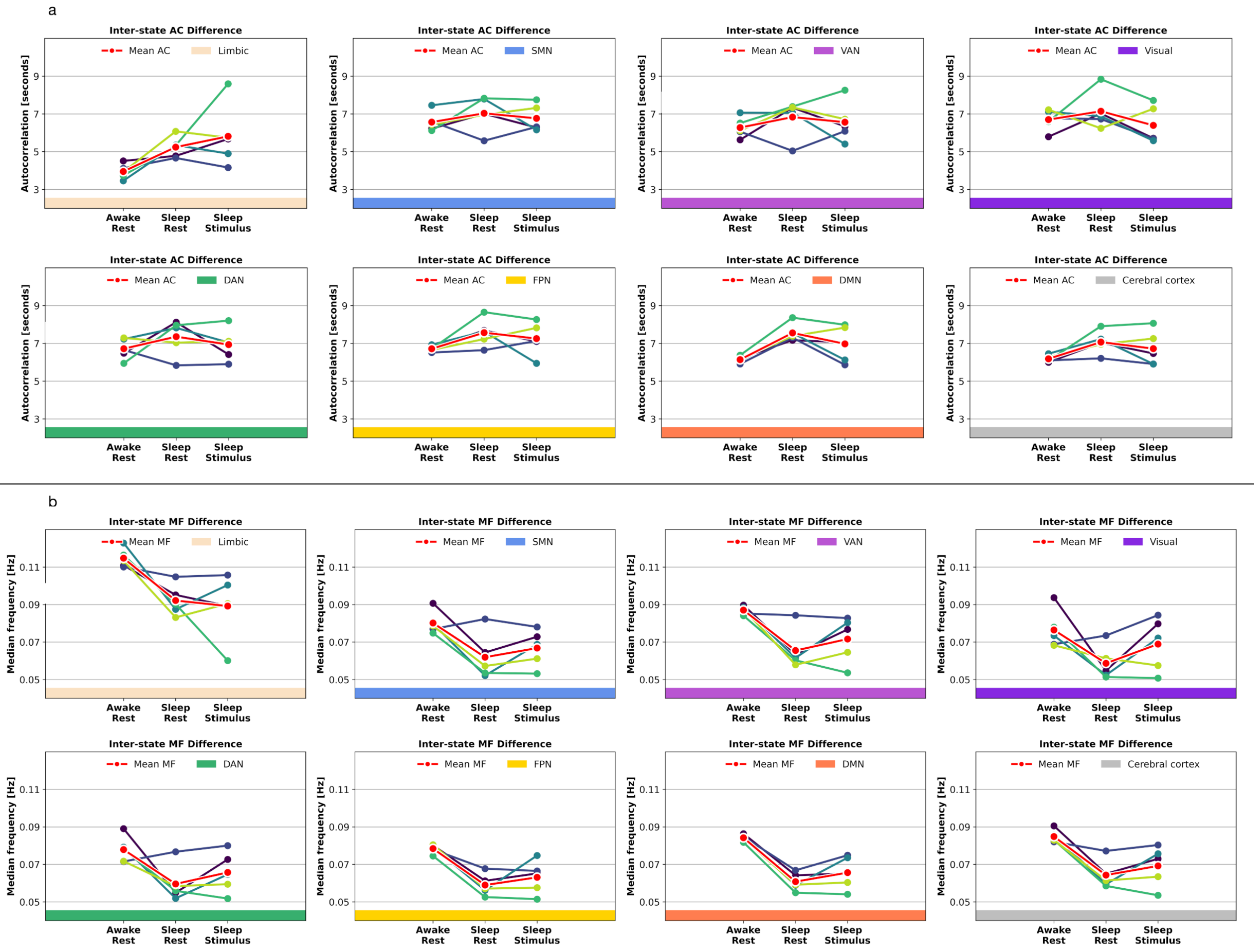

Supplementary Figure 2. Control analysis of four subjects fully asleep during the auditory stimulus state. Each line represents one subject, and the thicker red line represents the mean across subjects. **a)** Temporal autocorrelation (AC) results for the seven networks and cerebral cortex. **b)** Median frequency (MF) results for the seven networks and cerebral cortex. (Statistics, Student's paired *t*-Test; significance asterisks, $p < 0.05$ *, $p < 0.01$ ** , $p < 0.001$ ***; Bonferroni correction = p-values multiplied by three; $n = 4$ subjects.)

Supplementary Table 2
Temporal autocorrelation (AC) results for four subjects fully asleep during the auditory stimulus presentation

	Limbic	SMN	VAN	Visual	DAN	FPN	DMN	Cerebral cortex
Awake-Rest	3.90	6.55	6.19	6.91	6.84	6.76	6.28	6.25
Sleep-Rest	5.14	6.70	6.62	7.35	7.18	7.64	7.48	6.99
Sleep-Stimulus	4.71	5.85	5.37	5.17	5.67	6.01	5.96	5.61

Data represents voxel-based mean values across subjects of the AC in seconds. $n = 4$ subjects. (SMN, somatomotor network; VAN, ventral attention network; DAN, dorsal attention network; FPN, frontoparietal network; DMN, default-mode network.)

Supplementary Table 3
Temporal autocorrelation (AC) statistics for four subjects fully asleep during the auditory stimulus presentation

		Limbic	SMN	VAN	Visual	DAN	FPN	DMN	Cerebral cortex
Awake vs. Sleep-Rest	*t*-value	-3.45	-1.06	-1.15	-0.77	-1.18	-2.83	-8.37	-3.17
	p-value	0.039 *	0.522	0.471	0.726	0.454	0.071	0.002 **	0.051
Sleep-Rest vs. Sleep-Stimulus	*t*-value	-0.79	0.64	0.51	1.67	1.14	0.76	1.56	1.19
	p-value	2.293	0.833	0.953	0.256	0.475	0.733	0.290	0.448
Awake vs. Sleep-Stimulus	*t*-value	-2.31	-0.40	-0.51	0.66	-0.41	-1.24	-2.26	-1.21
	p-value	0.245	2.136	1.908	1.641	2.103	0.849	0.259	0.881

Statistics, Student's paired *t*-Test; significance asterisks, $p < 0.05$ *, $p < 0.01$ **, $p < 0.001$ ***; Bonferroni correction = *p*-values multiplied by three; $n = 4$ subjects. (SMN, somatomotor network; VAN, ventral attention network; DAN, dorsal attention network; FPN, frontoparietal network; DMN, default-mode network.)

Supplementary Table 4
Median frequency (MF) results for four subjects fully asleep during the auditory stimulus presentation

	Limbic	SMN	VAN	Visual	DAN	FPN	DMN	Cerebral cortex
Awake-Rest	0.114	0.077	0.086	0.071	0.073	0.078	0.083	0.083
Sleep-Rest	0.095	0.065	0.068	0.063	0.064	0.060	0.064	0.067
Sleep-Stimulus	0.101	0.077	0.083	0.083	0.079	0.075	0.076	0.081

Data represents voxel-based mean values across subjects of the MF in Hz. $n = 4$ subjects. (SMN, somatomotor network; VAN, ventral attention network; DAN, dorsal attention network; FPN, frontoparietal network; DMN, default-mode network.)

Supplementary Table 5
Median frequency (MF) statistics for four subjects fully asleep during the auditory stimulus presentation

		Limbic	SMN	VAN	Visual	DAN	FPN	DMN	Cerebral cortex
Awake-Rest vs. Sleep-Rest	*t*-value	4.18	3.02	4.11	2.34	2.69	7.95	14.84	5.13
	p-value	0.021 *	0.059	0.022 *	0.119	0.082	0.002 **	< 0.001 ***	0.01 *
Sleep-Rest vs. Sleep-Stimulus	*t*-value	0.40	-1.35	-1.31	-1.84	-1.54	-1.11	-1.67	-1.39
	p-value	1.931	0.373	0.389	0.210	0.297	0.494	0.256	0.355
Awake vs. Sleep-Stimulus	*t*-value	3.02	3.33	3.05	1.06	2.14	4.33	5.24	3.3520
	p-value	0.117	0.087	0.114	1.000	0.296	0.037 *	0.019 *	0.086

Statistics, Student's paired *t*-Test; significance asterisks, $p < 0.05$ *, $p < 0.01$ **, $p < 0.001$ ***; Bonferroni correction = *p*-values multiplied by three; $n = 4$ subjects. (SMN, somatomotor network; VAN, ventral attention network; DAN, dorsal attention network; FPN, frontoparietal network; DMN, default-mode network.)

Supplementary note 3: Additional simulations with different input scenarios

In the biophysical model, our initial choice of 25 Hz sine wave as input was arbitrary. In order to explore different input scenarios, we performed the following simulations where more complex inputs were presented to the model: (1) Combination of 3 sine waves with frequencies 25, 50, 75 Hz added on top of usual white noise input; (2) combination of 5 sine waves with frequencies 5, 25, 50, 75 and 100 Hz added on top of usual white noise input; and (3) a waveform that goes between 20 and 100 Hz over the course of

whole simulation added on top of white noise input. The results for scenario 1 and 2 are shown in Supplementary Figures 3 and 4. A part of the waveform of scenario 3 and the simulation results are shown in Supplementary Figures 5 and 6. These results are in agreement with the results we see in Figure 7 of the main manuscript.

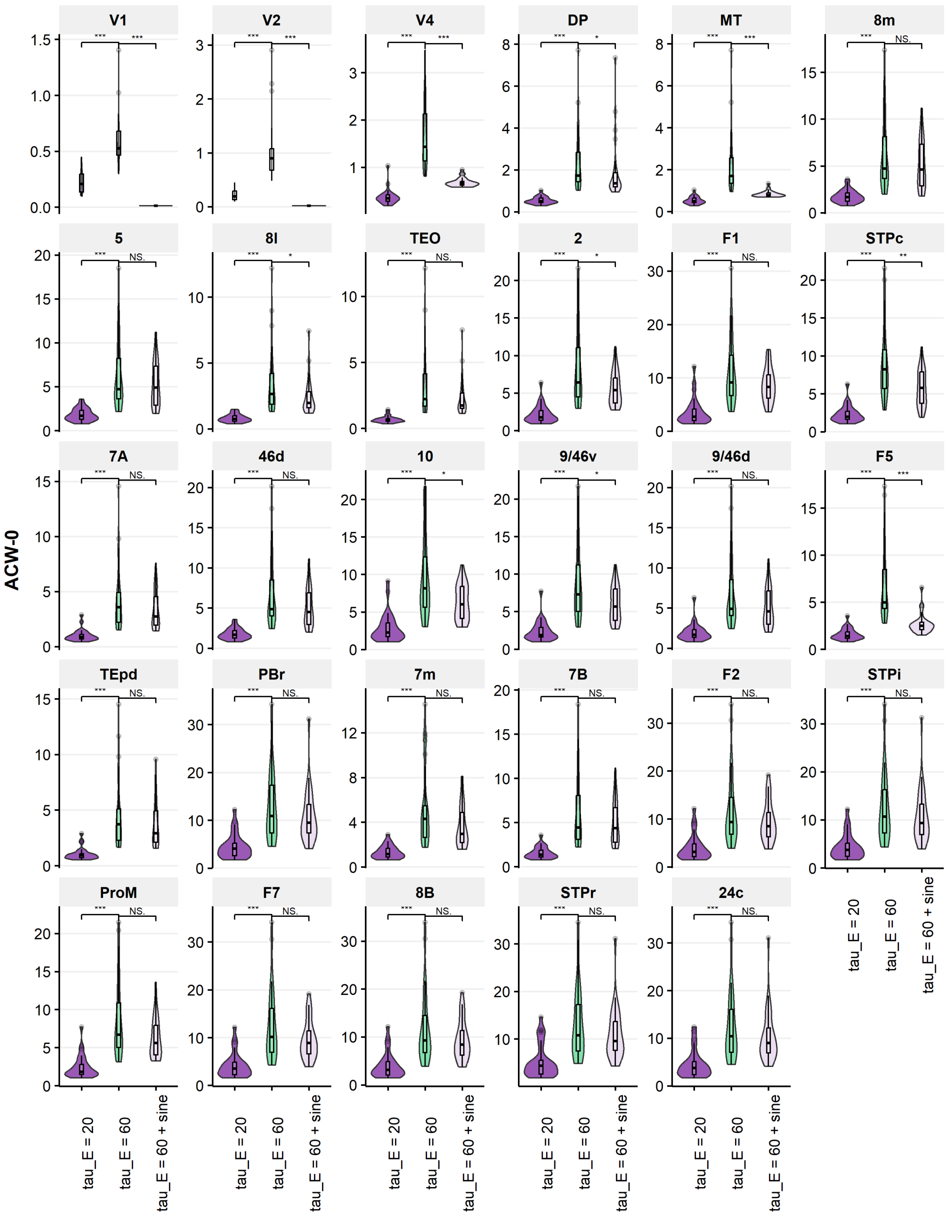

Supplementary Figure 3. Simulations performed with 3 sine waves with frequencies 25, 50, 75 Hz added on top of white noise input. The simulations were performed 30 times, the results show the distribution. Boxplot center point represents the median, boxes the interquartile range (IQR), and whiskers 1.5x IQR.

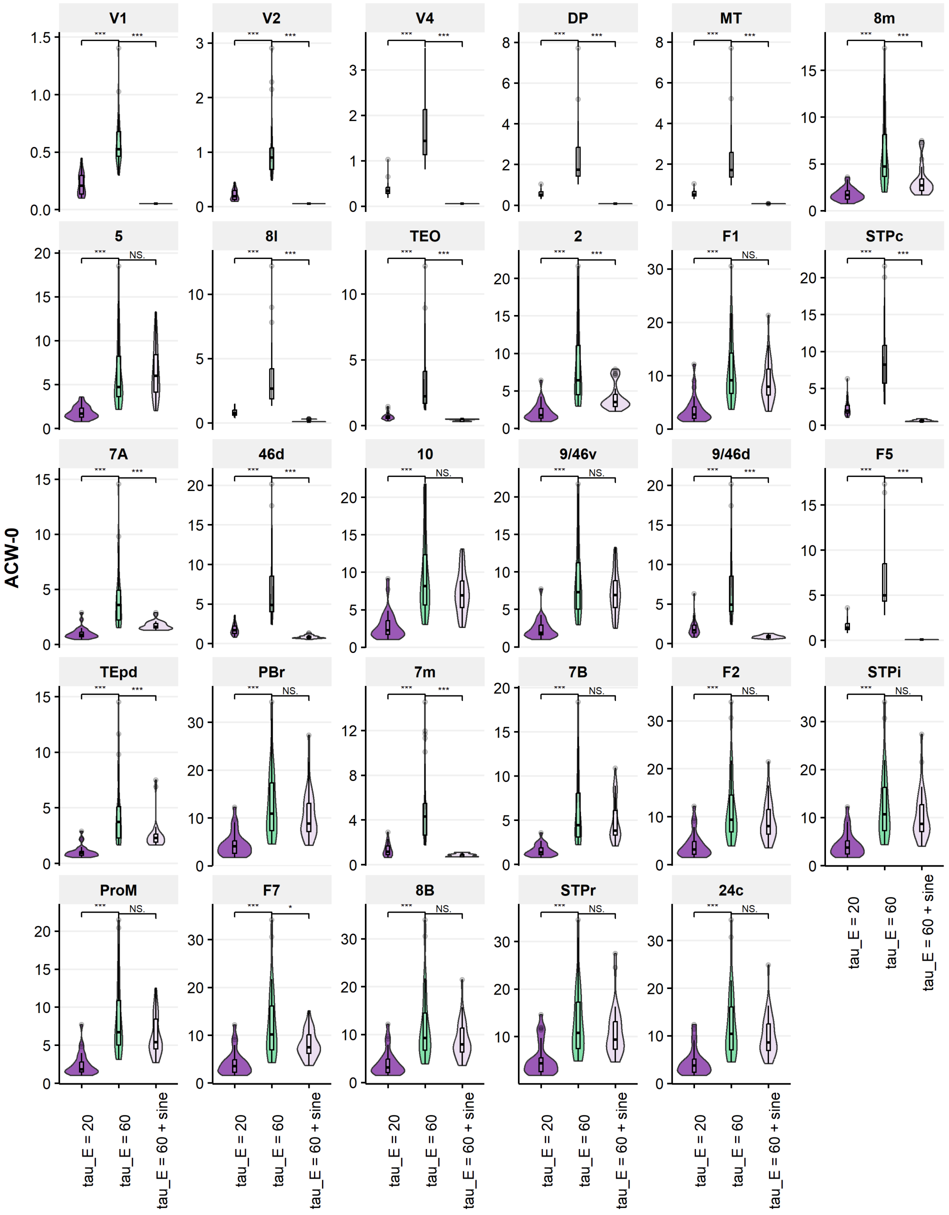

Supplementary Figure 4. Simulations performed with 3 sine waves with frequencies 5, 25, 50, 75, 100 Hz added on top of white noise input. The simulations were performed 30 times, the results show the distribution. Boxplot center point represents the median, boxes the interquartile range (IQR), and whiskers 1.5x IQR.

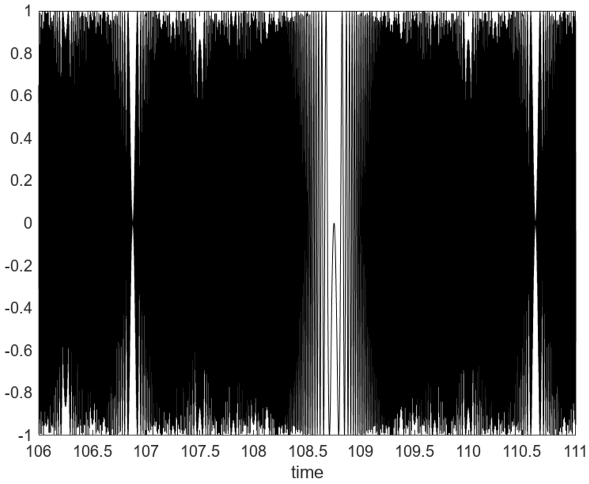

Supplementary Figure 5. The waveform that changes its frequency between 20 and 100 Hz over the simulation.

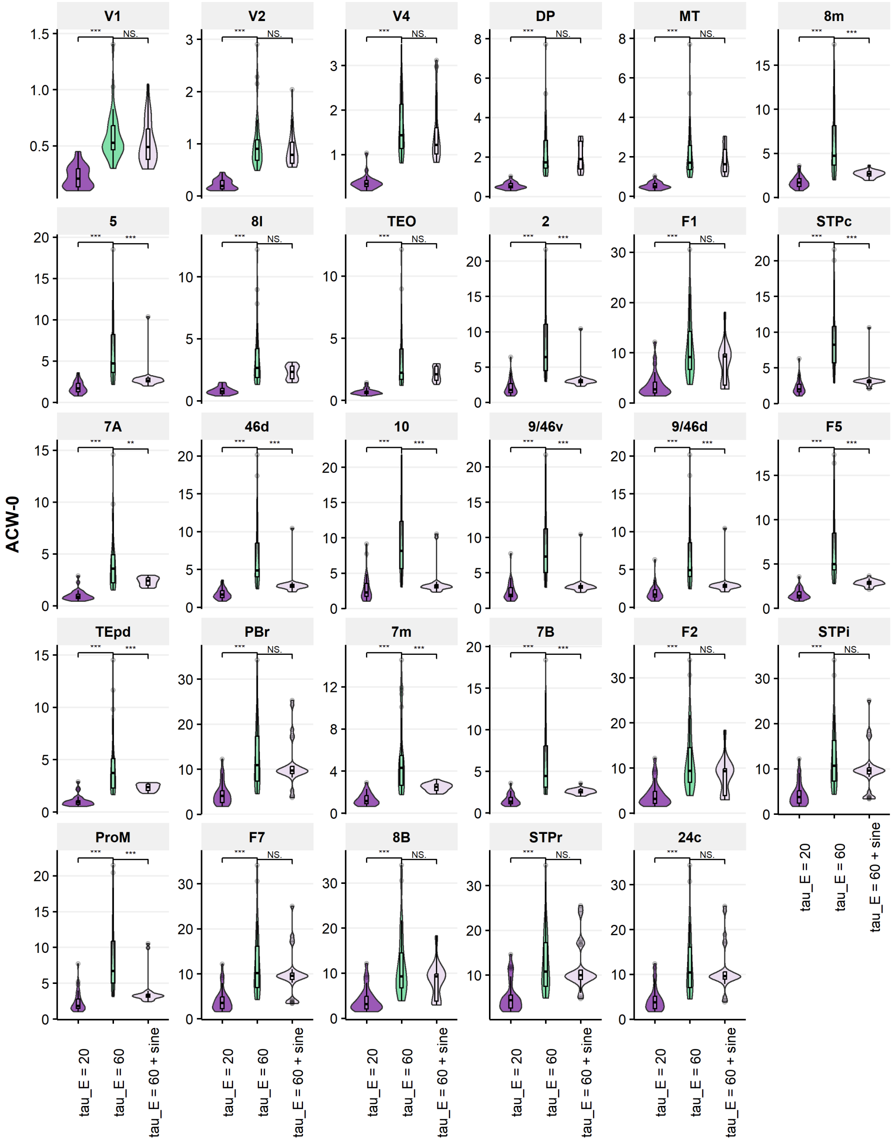

Supplementary Figure 6. Simulations performed with a waveform that changes its frequency between 20 and 100 Hz over the simulation added on top of white noise input. The simulations were performed 30 times, the results show the distribution. Boxplot center point represents the median, boxes the interquartile range (IQR), and whiskers 1.5x IQR.

Supplementary note 4: Results in Tables

Supplementary Table 6
Temporal autocorrelation (AC) results

	Limbic	SMN	VAN	Visual	DAN	FPN	DMN	Cerebral cortex
Awake-Rest	3.69	6.07	5.73	6.23	6.42	6.25	6.01	5.83
Sleep-Rest	4.89	6.89	6.54	7.26	7.39	7.22	7.05	6.82
Sleep-Stimulus	5.14	6.11	5.96	5.83	6.12	6.40	6.27	6.03

Data represents voxel-based mean values across participants of the AC in seconds. n = 17 participants. (SMN, somatomotor network; VAN, ventral attention network; DAN, dorsal attention network; FPN, frontoparietal network; DMN, default-mode network.)

Supplementary Table 7
Temporal autocorrelation (AC) statistics

		Limbic	SMN	VAN	Visual	DAN	FPN	DMN	Cerebral cortex
Awake-Rest vs. Sleep-Rest	t-value	-8.16	-3.68	-3.03	-2.99	-2.84	-5.63	-5.31	-5.89
	p-value	< 0.001 ***	0.002 **	0.008 **	0.009 **	0.012 *	< 0.001 ***	< 0.001 ***	< 0.001 ***
Sleep-Rest vs. Sleep-Stimulus	t-value	-0.87	2.04	1.52	3.97	3.27	2.33	2.94	2.75
	p-value	1	0.059	0.148	0.001 **	0.005 **	0.033 *	0.01 *	0.014 *

Statistics, Student's paired t-Test; significance asterisks, $p < 0.05$ *, $p < 0.01$ **, $p < 0.001$ ***; Bonferroni correction = p-values multiplied by two; n = 17 participants. (SMN, somatomotor network; VAN, ventral attention network; DAN, dorsal attention network; FPN, frontoparietal network; DMN, default-mode network.)

Supplementary Table 8
Median frequency (MF) results

	Limbic	SMN	VAN	Visual	DAN	FPN	DMN	Cerebral cortex
Awake-Rest	0.117	0.085	0.089	0.080	0.079	0.081	0.085	0.087
Sleep-Rest	0.098	0.066	0.070	0.060	0.062	0.064	0.068	0.069
Sleep-Stimulus	0.096	0.075	0.078	0.078	0.075	0.072	0.073	0.077

Data represents voxel-based mean values across participants of the MF in Hz. n = 17 participants. (SMN, somatomotor network; VAN, ventral attention network; DAN, dorsal attention network; FPN, frontoparietal network; DMN, default-mode network.)

Supplementary Table 9
Median frequency (MF) statistics

		Limbic	SMN	VAN	Visual	DAN	FPN	DMN	Cerebral cortex
Awake-Rest vs. Sleep-Rest	*t*-value	9.06	6.27	8.33	4.91	5.32	11.67	9.87	9.20
	p-value	< 0.001 ***	< 0.001 ***	< 0.001 ***	< 0.001 ***	< 0.001 ***	< 0.001 ***	< 0.001 ***	< 0.001 ***
Sleep-Rest vs. Sleep-Stimulus	*t*-value	0.70	-3.32	-3.14	-5.21	-5.02	-3.47	-3.46	-4.14
	p-value	1	0.004 **	0.006 **	< 0.001 ***	< 0.001 ***	0.003 **	0.003 **	< 0.001 ***

Statistics, Student's paired *t*-Test; significance asterisks, $p < 0.05$ *, $p < 0.01$ **, $p < 0.001$ ***; Bonferroni correction = *p*-values multiplied by two; $n = 17$ participants. (SMN, somatomotor network; VAN, ventral attention network; DAN, dorsal attention network; FPN, frontoparietal network; DMN, default-mode network.)

Supplementary Table 10
Topographic similarity of temporal autocorrelation (AC) results

		Limbic	SMN	VAN	Visual	DAN	FPN	DMN	Cerebral cortex
Awake-Rest		0.15	0.36	0.29	0.65	0.52	0.22	0.28	0.26
Sleep-Rest		0.34	0.31	0.33	0.30	0.26	0.18	0.15	0.12
Sleep-Stimulus		0.76	0.60	0.60	0.86	0.68	0.59	0.52	0.55
Sleep-Rest vs. Sleep-Stimulus	*t*-value	-42.71	-107.5	-58.26	-163.2	-93.75	-102.38	-144.46	-786.33
	p-value	< 0.001 ***	0 ***	0 ***	0 ***	0 ***	0 ***	0 ***	0 ***

Data represents the median of voxel-based pairwise Pearson correlation (Fisher Z transformed) across participants of the AC. Statistics, Student's paired *t*-Test for Pearson correlations; significance asterisks, $p < 0.05$ *, $p < 0.01$ **, $p < 0.001$ ***; $n = 17$ participants. (SMN, somatomotor network; VAN, ventral attention network; DAN, dorsal attention network; FPN, frontoparietal network; DMN, default-mode network.)

Supplementary Table 11
Topographic similarity of median frequency (MF) results

		Limbic	SMN	VAN	Visual	DAN	FPN	DMN	Cerebral cortex
Awake-Rest		0.19	0.59	0.20	0.78	0.46	0.07	0.12	0.23
Sleep-Rest		0.43	0.55	0.45	0.33	0.46	0.25	0.24	0.26
Sleep-Stimulus		0.81	0.72	0.55	1.04	0.70	0.54	0.47	0.53
Sleep-Rest vs. Sleep-Stimulus	*t*-value	-43.92	-54.03	-26.43	-222.73	-60	-82.93	-107	-561.68
	p-value	< 0.001 ***	0 ***	< 0.001 ***	0 ***	0 ***	0 ***	0 ***	0 ***

Data represents the median of voxel-based pairwise Pearson correlation (Fisher Z transformed) across participants of the MF. Statistics, Student's paired *t*-Test for Pearson correlations; significance asterisks, $p < 0.05$ *, $p < 0.01$ **, $p < 0.001$ ***; $n = 17$ participants. (SMN, somatomotor network; VAN, ventral attention network; DAN, dorsal attention network; FPN, frontoparietal network; DMN, default-mode network.)
