# Supplementary material for: Auditory inputs modulate intrinsic neuronal timescales during sleep
Source: Commun Biol. 2023 Nov 20;6:1180. doi: 10.1038/s42003-023-05566-8 (PMC10661171; doi:10.1038/s42003-023-05566-8)
Supplement: Supplementary file 2 — Description of Additional Supplementary Files [file 42003_2023_5566_MOESM2_ESM.docx]

Description of Additional Supplementary Files

**File name:** Supplementary data 1

**Description:** This Excel sheet includes the source data for Figures 2, 3, 4, 5, 6, and 7.
